# Integrated metaheuristic algorithms with extreme learning machine models for river streamflow prediction

**DOI:** 10.1038/s41598-024-63908-w

**Published:** 2024-06-12

**Authors:** Nguyen Van Thieu, Ngoc Hung Nguyen, Mohsen Sherif, Ahmed El-Shafie, Ali Najah Ahmed

**Affiliations:** 1 Faculty of Computer Science, PHENIKAA University, Yen Nghia, Ha Dong, Hanoi, 12116 Viet Nam; 2 Artificial Intelligence Independent Research Group, Hanoi, Viet Nam; 3 Civil and Environmental Engineering Department, College of Engineering, United Arab Emirates University, P.O. Box 15551, Al Ain, United Arab Emirates; 4 Department of Civil Engineering, Faculty of Engineering, University of Malaya (UM), 50603, Kuala Lumpur, Malaysia; 5 National Water and Energy Center, United Arab Emirate University, P.O. Box 15551 Al Ain, United Arab Emirates; 6 Department of Engineering, School of Engineering and Technology, Sunway University, No. 5, Jalan Universiti, Bandar Sunway, 47500 Selangor Darul Ehsan Malaysia

**Keywords:** River streamflow, Forecasting model, Extreme learning machine, Metaheuristic optimization algorithm, Nature-inspired algorithms, Nile river, Environmental sciences, Hydrology, Mathematics and computing, Computational science, Computer science, Information technology

## Abstract

Accurate river streamflow prediction is pivotal for effective resource planning and flood risk management. Traditional river streamflow forecasting models encounter challenges such as nonlinearity, stochastic behavior, and convergence reliability. To overcome these, we introduce novel hybrid models that combine extreme learning machines (ELM) with cutting-edge mathematical inspired metaheuristic optimization algorithms, including Pareto-like sequential sampling (PSS), weighted mean of vectors (INFO), and the Runge–Kutta optimizer (RUN). Our comparative assessment includes 20 hybrid models across eight metaheuristic categories, using streamflow data from the Aswan High Dam on the Nile River. Our findings highlight the superior performance of mathematically based models, which demonstrate enhanced predictive accuracy, robust convergence, and sustained stability. Specifically, the PSS-ELM model achieves superior performance with a root mean square error of 2.0667, a Pearson’s correlation index (R) of 0.9374, and a Nash–Sutcliffe efficiency (NSE) of 0.8642. Additionally, INFO-ELM and RUN-ELM models exhibit robust convergence with mean absolute percentage errors of 15.21% and 15.28% respectively, a mean absolute errors of 1.2145 and 1.2105, and high Kling-Gupta efficiencies values of 0.9113 and 0.9124, respectively. These findings suggest that the adoption of our proposed models significantly enhances water management strategies and reduces any risks.

## Introduction

River streamflow is a fundamental hydrological parameter pivotal for the effective planning of water resource projects, hydropower generation systems, agricultural practices, and irrigation schedules. In essence, it facilitates the optimal utilization of existing water resources^1^. Hence, river streamflow forecasting has garnered substantial interest, not only within the domain of hydrology but also among economists, decision-makers, and policymakers. This collective focus aims to attain the optimal operational management of river basin systems^2^. Timely and precise data on river streamflow holds particular significance, notably aiding in the regulation of reservoir outflows during periods of low streamflow and offering advance alerts for impending or potential flood events. From an economic standpoint, accurate forecasting of river streamflow serves as a pivotal factor in optimizing hydropower generation and meeting agricultural water demands^3^. In addition, providing decision-makers with precise river-streamflow forecasting information could aid in the accurate assessment of flood magnitude and prevent casualties and limitless damage to property and infrastructure^4^. In fact, it is difficult to create a completely safe system against flood events, but with good forecasts for river-streamflow. In this context, developing river-streamflow forecasts is continuously considered a high priority for hydrologists to investigate the potential of different methods and approaches to achieve acceptable forecasting accuracy^5^. Indeed, deciphering the temporal patterns of river streamflow across various time intervals is a formidable challenge owing to its pronounced variance, stochastic nature, and nonlinear characteristics. Throughout the latter half of the twentieth century, statistical modeling approaches emerged as valuable tools for river streamflow forecasting. Among these methods, the autoregressive integrated moving average (ARIMA) and seasonal autoregressive integrated moving average (SARIMA) methods have garnered widespread adoption for this purpose^6^. Basically, statistical methods conceptually attempt to linearly identify the intrinsic interrelationship within the historical actual river streamflow data. Hence, nonlinearity and stochastic patterns, which are the main features of river streamflow, were not detected^7^. The application of statistical methodologies has encountered a significant limitation in effectively predicting abrupt shifts in river streamflow. To address this shortcoming inherent in statistical approaches, alternative methods such as the Kalman filtering (KF) model have been introduced. The KF model offers a solution by explicitly incorporating all conceivable sources of uncertainty, thereby mitigating the challenges encountered during simulation^8^. Although KF has proven to be a well-known mathematical tool often used for stochastic prediction, the development of standard KF models for forecasting hydrological parameters has shown drawbacks in handling the highly stochastic patterns of river streamflow and has provided poor forecasting accuracy for extreme river streamflow^9^. In addition, the main challenge related to adopting the KF for river-streamflow forecasting is the need for an accurate predefined stochastic model for all their input variables^10^. Furthermore, when using many parameters as inputs for the model, there must be information regarding the values of the covariance of the river streamflow as well as the other parameters used, such as the pattern of rainfall, which requires a comprehensive preprocessing analysis for all the input variables used in the model structure.

Since the beginning of the twenty-first century, a new modeling direction for the development of river-streamflow forecasting modeling techniques based on artificial intelligence (AI) and machine learning (ML) has been introduced to overcome the drawbacks of statistical and KF methods^11–14^. Although these AI and ML methods showed outstanding performance over the statistical and KF methods, they experienced other types of drawbacks during the development of the model, such as the best model’s input selection, overfitting, and achieving the optimal values of the hyperparameters^15^. Further enhancements of these models have also been introduced to overcome these drawbacks. For example, AI or ML models can be integrated with preprocessing methods, such as normalizing the data or applying a wavelet to denoise white noise, which could inherently exist in the data used to equip the model with a better shape of the data before modeling^16^. Moreover, several techniques, such as the regularization approach, have been used to overcome the overfitting problem. Furthermore, AI and ML models have been integrated with advanced optimization algorithms, such as the genetic algorithm (GA) and other nature-inspired optimization algorithms, to ensure optimal model input selection^17–19^, such as particle swarm optimization, bacterial foraging optimization, and ant bee colony, to avoid trapping in local minima and ensure stable convergence to find the optimal values of the hyperparameters^20–22^. Although all of these enhancements improved the ability of the AI and ML methods to overcome most of the modeling problems, the primary issue with the AI and ML models was finding the ideal values for the hyperparameters through trial-and-error procedures and choosing the proper optimization algorithms to ensure stability in the convergence procedure during model training. In this context, the limitations of river-streamflow forecasting models using statistical, KF, AI, and ML-based methods have necessitated the search for other predominantly AI-based methods.

The extreme learning machine (ELM) is an innovative learning architecture that has gained popularity in fields such as multiresolution signal processing, large-scale computing, and intelligent modeling^23^. The extreme learning machine (ELM) represents a set of machine learning methodologies designed to eliminate the necessity of adjusting hidden neurons, bridging the gap between conventional biological training and intelligent training framework mechanisms. In accordance with the algorithmic framework and principles of the ELM, the notion of random hidden neurons embodies diverse learning framework mechanisms, ensuring learning efficacy independent of neural processing capacity. Consequently, the ELM has significant advantages over traditional neural networks and support vector machines and is characterized by swift learning, straightforward implementation, and minimal human intervention. Notably, ELMs have garnered widespread application owing to their exceptional generalization performance and operational efficiency^24^. Numerous enhanced ELM algorithms have been presented along with an expansion in the range of supervised^25^, semisupervised^26^, and unsupervised learning^27^ methods that can be used to execute the standard ELM^28^.

Particularly, ELM-based models have found widespread application in hydrology-related fields. Atiquzzaman and Kandasamy^29^ used ELM to predict hydrological time-series data from the Tryggevaelde Basin in Denmark and the Mississippi River near Vicksburg, USA. Their findings suggested that ELM outperformed Artificial Neural Networks (ANN) and EC-SVM in metrics such as Root Mean Square Error (RMSE) and Normalized RMSE (NRMSE). Wang^30^ proposed several ELMs for hydrological drought forecasting, proving that the self-adaptive differential evolution ELM outperforms conventional ELM and Support Vector Machine (SVM) in error reduction. Yaseen^31^ developed an enhanced version of ELM (EELM) for river flow predicting, demonstrating its superiority over Support Vector Regression (SVR) and ELM across multiple metrics including Nash–Sutcliffe Efficiency (NSE), Willmott’s Index (WI), Pearson correlation coefficient (R), Mean Absolute Error (MAE), and RMSE. Wang^32^ proposed a model named TVF-EMD-SSA-ELM, which combines time-varying filtering (TVF) based empirical mode decomposition (EMD), the salp swarm algorithm (SSA), and extreme learning machine (ELM). This model was applied to the monthly runoff prediction for Manwan Hydropower, Hongjiadu Hydropower, and Yingluoxia Hydrological Station. Their results showed that the SSA algorithm enhances the ELM model, yielding superior performance compared to back propagation (BP), ELM, and PSO-ELM algorithms in metrics such as NRMSE) and MAPE.

Despite its wide adoption, ELM has certain drawbacks, such as classic ELM struggles to effectively handle highly nonlinear and stochastic data due to its reliance on random initialization of the input-to-hidden layer weights^33^. While the stochastic nature of extreme learning machines (ELMs) increases their efficiency, it also brings sensitivity to initialization parameters, which may affect the model's performance and generalization capabilities. Unlike iterative methods, ELMs randomly initialize weights and analytically determine output weights, which may limit their ability to reach optimal solutions, especially in complicated or nonlinear problems. To address these problems, in this paper, we introduce three state-of-the-art mathematical-inspired metaheuristic algorithms for training the ELM network: Pareto-like sequential sampling (PSS), weighted mean of vectors (INFO), and the Runge–Kutta optimizer (RUN). The rationale for adopting these three recommended algorithms derives from the theoretical idea of the No-Free Lunch theorem^34^, which states that no single algorithm outperforms all other algorithms on all problems. Thus, proposing three algorithms is expected to yield superior effectiveness and performance capabilities.

- PSS^35^ is a recently proposed algorithm that has shown strong performance in a variety of engineering design problems^36^. PSS, which employs techniques such as Latin hypercube and Monte Carlo sampling, demonstrates significant exploration capabilities, making it a great choice for utilization in hydrology.
- INFO, proposed in 2022 and referenced over 400 times^37^, has exhibited remarkable capability across diverse fields and applications^38^. With its ability to balance exploration and exploitation phases effectively, INFO is highly efficient, thus motivating us to apply it in domains with complex problems like river streamflow forecasting.
- RUN^39^, another recently proposed algorithm, adheres to the metaphor-free population-based criterion and is inspired by the well-known Runge–Kutta technique in mathematics. This algorithm has been shown to be highly efficient in a variety of real-world issues and application^40–42^. As a result, implementing RUN in areas with complicated and difficult problems, such as hydrology, is considered crucial.

With all of the aforementioned reasons and the additional goal of this paper being to develop effective river streamflow forecasting models. The proposed models are extensively tested by applying it to historical river streamflow data collected from the main gauge station at Egypt's Aswan High Dam (AHD). This station is selected specifically for its extensive monthly record spanning 130 years on the Nile River, which is the largest and longest river basin globally.

The proposed algorithms, when combined with the ELM network, including PSS, INFO, and RUN, will be compared with 17 other algorithms from eight different types of metaheuristic optimization: swarm-inspired, evolutionary-inspired, physics-inspired, human-inspired, biology-inspired, system-inspired, music-inspired, and math-inspired. The fundamental reason for using numerous models in such comparisons is to objectively evaluate the capabilities of the proposed models. This is especially important given the No Free Lunch theory, which states that no algorithm outperforms all others in all problem categories. As a result, this comparison is critical to ensuring the study's comprehensiveness. Furthermore, the performance of these models will be evaluated using six performance metrics: mean absolute error (MAE), root mean square error (RMSE), Pearson's correlation coefficient (R), mean absolute percentage error (MAPE), Nash–Sutcliffe efficiency (NSE), and Kling-Gupta efficiency (KGE). These are common metrics often employed in fields related to streamflow and hydrology.

The remainder of this article is structured as follows. Section "Materials and Methods" describes the data collection, the case studies, the theoretical background of the proposed optimizers (PSS, INFO, and RUN), the extreme learning machine, and our proposed hybrid models. Section "Experiments" describes details the comparison models, parameter settings, and performance metrics. Section "Results and Discussion" presents the achieved results, an analysis of the findings, and discussions about the model’s performance. Section "Limitations and future directions" highlights potential limitations and provides recommendations for future research based on this study. Finally, Section "Conclusion" summarizes our study and outlines our future works.

## Materials and methods

### Study area and data acquisition

**Figure 1:**
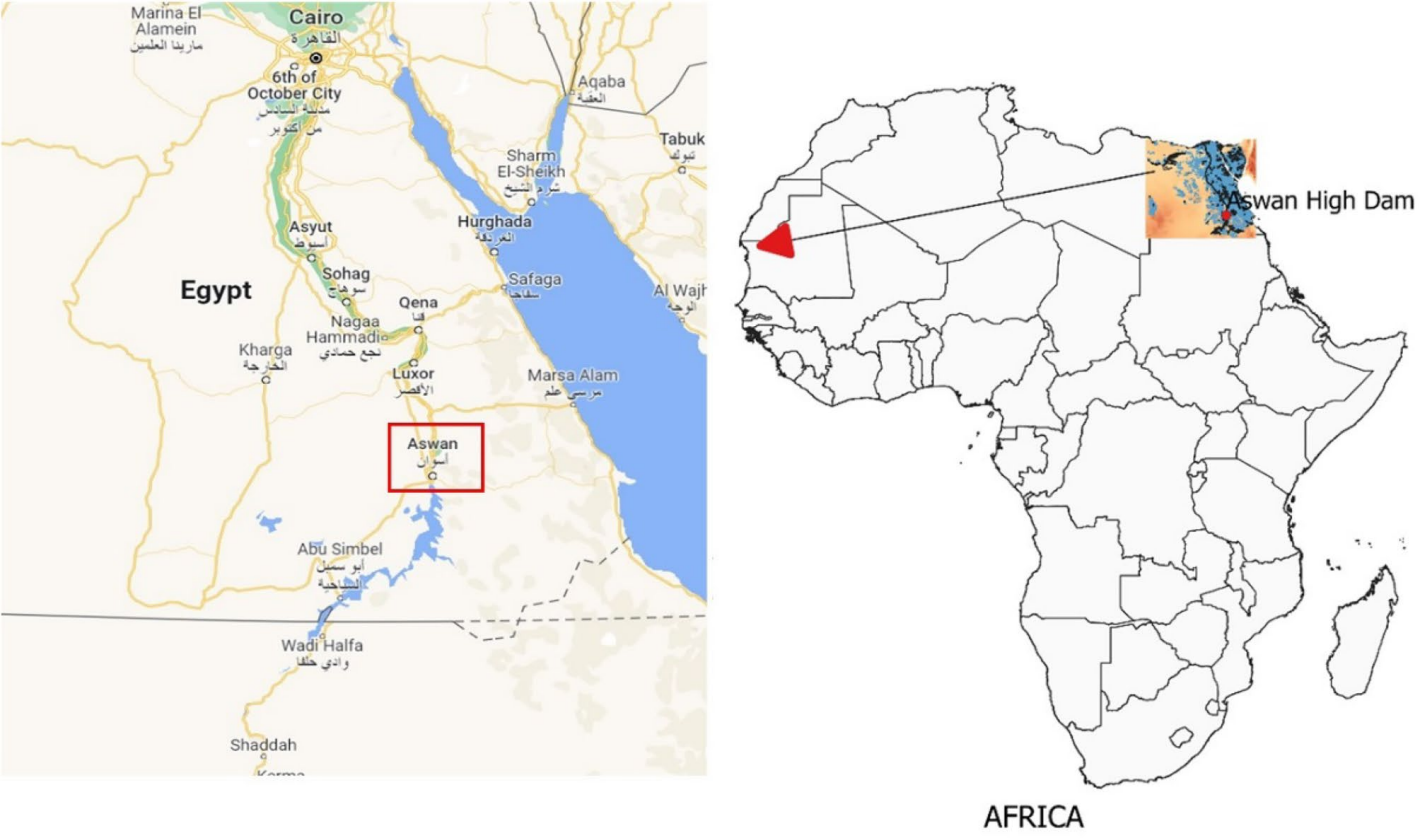
The location of the case study Aswan High Dam (AHD).

**Figure 2:**
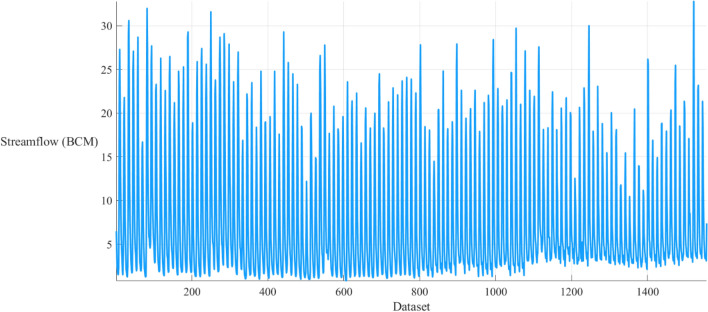
Natural monthly streamflow of the Nile River in the BCM.

The main objective of this study is to forecast the natural flow pattern of the Nile River at the AHD site, which is located in the southern region of Egypt, as depicted in Fig. 1. The AHD is a large reservoir constructed in southern Egypt by damming the river. Its construction aimed to control flooding, generate hydroelectricity, and provide irrigation water. This study uses natural flow data records spanning 130 years (from 1870 to 2000) at the AHD as the basis for the dataset^43^. This research utilized the Nile River inflow data from Aswan, as published by the Egyptian Ministry of Water Resources and Irrigation. This dataset is one of the most extensive and long-lasting of its kind, providing valuable insight into the long-term flow trends in the region. The fluctuating flow pattern is clearly depicted in Fig. 2, which illustrates the yearly flow.

### Model Descriptions

In this section, a full description of the proposed algorithms and models is provided. First, a clear representation of the foundation of the proposed algorithms, including the PSS, INFO, and RUN algorithms, is provided. Subsequently, comprehensive discussions of the ELM model for time-series modeling will be reported. Finally, the proposed hybrid model, which combines metaheuristic algorithms and an ELM network, is presented.

Before describing the details of the proposed algorithms, the initialization process, fitness function, and several symbols used throughout the three proposed algorithms are introduced below. All metaheuristic optimization algorithms involve an initialization process. This process starts by initializing N random solutions (population size or pop_size). Each solution is a D-dimensional vector (D variables or problem size) obtained using a uniform random distribution, which can be described by Eq. (1).

1 $$X_{i,j}^{0} = X_{j}^{min} + R_{i,j} .\left( {X_{j}^{max} - X_{j}^{min} } \right)$$

where i = 1,2… N; j = 1,2,…. D; $X_{i,j}^{0}$ is the initial position vector of the $i^{th}$ solution; $X_{j}^{min}$ and $X_{j}^{max}$ denote the minimum and maximum values, respectively, for the $j^{th}$ dimension of the $i^{th}$ solution; and $R_{i,j}$ is a uniform random value in the range of [0, 1].

The fitness function is a function that is used to evaluate the quality of a solution and can be composed of one or multiple objective functions or variations of objective functions. For minimization problems, the lower the fitness function is, the better the solution, and conversely, the higher the fitness function is, the worse the solution. Some of the standard notations used throughout this paper are presented in Table 1.

**Table 1:** The common mathematical notation used in this paper.

Symbol	Meaning
$X^{t}$	Position of solution at the $t\,th$ iteration (generation/epoch)
$X_{i, j}^{t}$	The value of the $j\,th$ dimension of $i\,th$ solution at the $t\,th$ iteration
$X_{r}$	The random solution (position)
$T_{max}$	The maximum number of iterations
$X_{best}^{g}$	The current best solution at $g\,th$ generation
$X_{best}$	The global best solution (The final solution of algorithm)
$fit_{best}^{t}$	The current best fitness value at $t\,th$ generation
$fit_{best}$	The global best fitness value
$g$, $g_{max}$	The current generation and maximum number of generations
$randn$	The random number generated from normal distribution

#### Pareto-like sequential sampling (PSS) optimization

**Figure 3:**
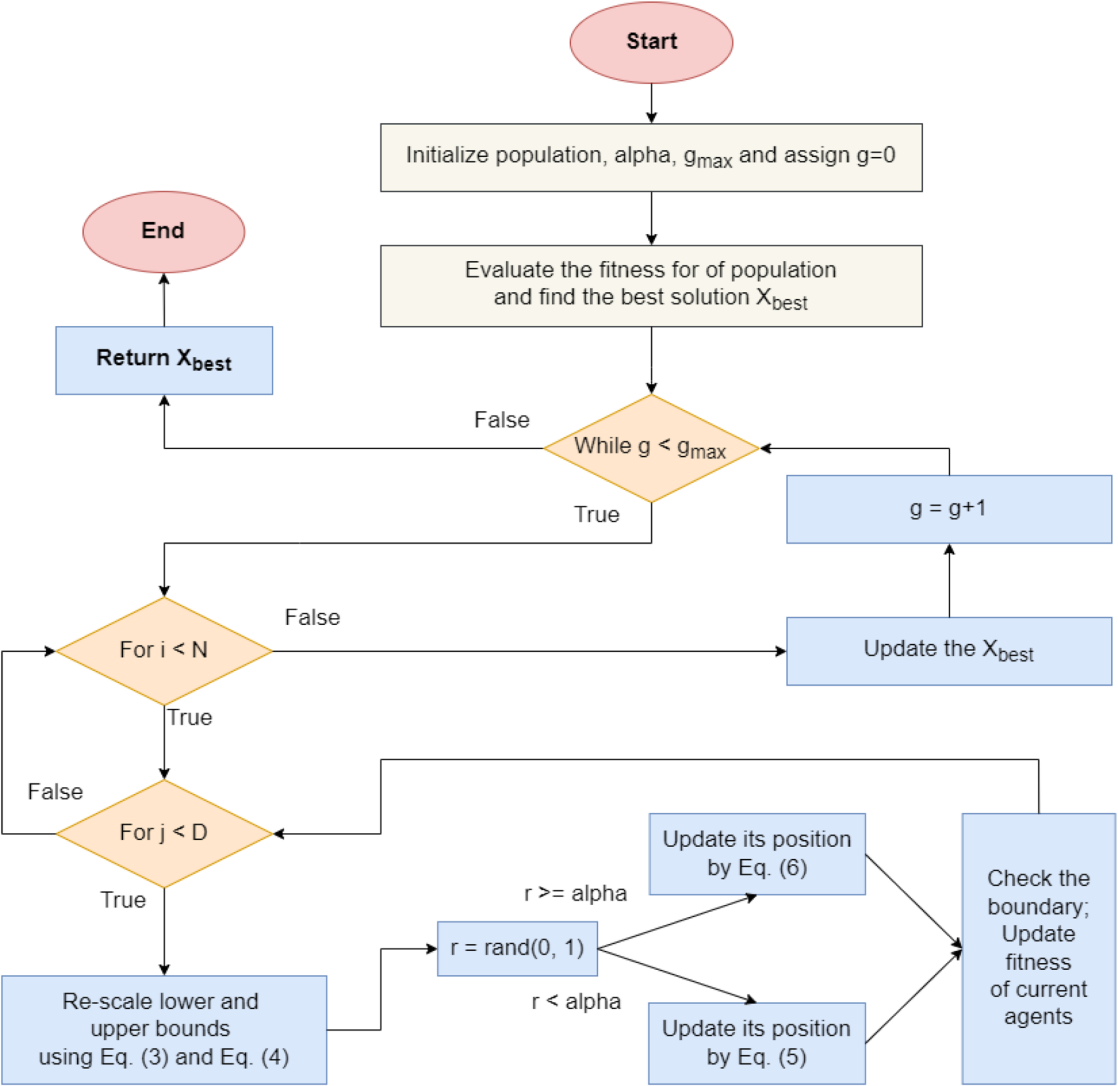
Pareto-like Sequential Sampling (PSS) Optimization.

The PSS algorithm is a recently proposed optimization algorithm^35^ based on simple sampling and design-of-experiment (DOE) methods, such as Monte Carlo and Latin hypercube sampling. Despite its simplicity, the PSS has been shown to outperform several recent algorithms (PFA, PSO, WOA, etc.) in benchmark functions and engineering problems^35^. The algorithm starts by initializing a set of N vectors with random coefficient vectors generated using DOE methods as modeled in Eq. (2)

2 $$X_{i, j} = X_{j}^{min} + R_{i,j} \odot \left( {X_{j}^{max} - X_{j}^{min} } \right)$$

where $X_{j}^{min}$ and $X_{j}^{max}$ are the lower bound and upper bound for the jth dimension, respectively; $R_{i,j}$ is the random variable generated using the DOE sampling method; and $\odot$ is the elementwise multiplication of vectors. After the initialization of the population, the PSS algorithm determines the best solution to rescale the lower and upper bounds for the prominent area (Eq. 3), which is then used to update the next positions of the solutions.

3 $$X_{j}^{min1} = X_{best, j} - \delta_{j} ; X_{j}^{max1} = X_{best, j} + \delta_{j}$$

4 $$\delta_{j} = \frac{1}{2}\left( {1 - \alpha } \right)\left( {1 - \frac{g}{{g_{max} }}} \right)\left( {X_{j}^{max} - X_{j}^{min} } \right)$$

where the acceptance probability α describes the resampling of the 80/20 analogy. After that, the PSS updates the position of the current search agent using either the prominent domain (with the new lower and upper bounds) or the overall domain (the default lower and upper bounds). This process can be modeled using Eqs. (5) and 6

5 $$X_{i,j}^{g + 1} = X_{j}^{min1} + \mu_{i,j } \odot \left( {X_{j}^{max1} - X_{j}^{min1} } \right)$$

6 $$X_{i,j}^{g + 1} = X_{j}^{min} + \mu_{i,j } \odot \left( {X_{j}^{max} - X_{j}^{min} } \right)$$

where $\mu_{i,j}$ is a random variable generated using the DOE sampling method and $\odot$ represents the elementwise multiplication of vectors. The flowchart of the PSS algorithm is shown in Fig. 3.

#### Weighted mean of vectors (INFO) optimization

The INFO algorithm is a recent math-inspired metaheuristic proposed by^37^. It consists of four key phases: initialization (described above), updating rule, vector combining, and local search. Due to its solid mathematical concepts, INFO has been demonstrated to be effective in several applications, such as engineering optimization^44^, image classification tasks^45^, and feature selection^46^. This section briefly overviews some of the main operators and equations used in INFO, with more detailed information available in^37^.

##### Updating rule phase

Generally, INFO generates a set of random vectors and then derives the weighted mean from these vectors instead of modifying the position of the current vector toward an optimal solution. The mean rule can be described as follows:

7 $$MeanRule = r1*WM1^{g} + \left( {1 - r1} \right)*WM2^{g}$$

8 $$WMt^{g} = \delta *\frac{{w_{1} \left( {X_{a1} - X_{a2} } \right) + w_{2} \left( {X_{a1} - X_{a3} } \right) + w_{3} \left( {X_{a2} - X_{a3} } \right)}}{{w_{1} + w_{2} + w_{3} + \varepsilon }} + \varepsilon *randn$$

9 $$w_{1} = \cos \left( {f\left( {X_{a1} } \right) - f\left( {X_{a2} } \right) + \pi } \right)*{\text{exp}}\left( { - \left| {\frac{{f\left( {X_{a1} } \right) - f\left( {X_{a2} } \right)}}{w}} \right|} \right)$$

10 $$w_{2} = \cos \left( {f\left( {X_{a1} } \right) - f\left( {X_{a3} } \right) + \pi } \right)*{\text{exp}}\left( { - \left| {\frac{{f\left( {X_{a1} } \right) - f\left( {X_{a3} } \right)}}{w}} \right|} \right)$$

11 $$w_{3} = \cos \left( {f\left( {X_{a2} } \right) - f\left( {X_{a3} } \right) + \pi } \right)*{\text{exp}}\left( { - \left| {\frac{{f\left( {X_{a2} } \right) - f\left( {X_{a3} } \right)}}{w}} \right|} \right)$$

12 $$\delta = \left( {2*r2 - 1} \right)*\beta = \left( {2*r2 - 1} \right)*2*{\text{exp}}\left( { - 4* \frac{g}{{g_{max} }}} \right)$$

where $g$ and $g_{max}$ represent the current generation (iteration/epoch) and maximum number of generations, respectively. $r1$ and $r2$ are random numbers generated from the ranges [0, 0.5] and [0, 1], respectively. $randn$ is a random number generated from a normal distribution. $\varepsilon$ is a very small number, $f()$ is the fitness function, and $t$ is the index of the WM. When $t = 1$, then $a1, a2$ and $a3$ are different intergers chosen randomly from the [1, N] range, and $w = \max \left( {f\left( {X_{a1} } \right), f(X_{a2} } \right), f(X_{a3} ))$. When $t = 2$, $X_{a1} = X_{best}$ represents the best solution, $X_{a2} = X_{better}$ represents the better solution, $X_{a3} = X_{worst}$ represents the worst solution in the current iteration, and $w = f\left( {X_{a3} } \right)$. After calculating $MeanRule$, INFO generates 2 new solutions $z1$ and $z2$ based on $X_{best}$, $X_{better}$, $X_{a1}$, and the current solution $X_{i}$. In the case of a random number $r3 < 0.5$, $z1$ and $z2$ are generated by Eq. (13). Otherwise, $z1$ and $z2$ are generated by Eq. (14).

13 $$\left\{ {\begin{array}{*{20}l} {z1 = X_{i} + \sigma *MeanRule + randn*\frac{{X_{best} - X_{a1} }}{{f\left( {X_{best} } \right) - f\left( {X_{a1} } \right) + 1 }}} \hfill \\ {z2 = X_{best} + \sigma *MeanRule + randn*\frac{{X_{a1} - X_{best} }}{{f\left( {X_{a1} } \right) - f\left( {X_{best} } \right) + 1 }}} \hfill \\ \end{array} } \right.\,\,\,\,\,\,\,if\,\,\,\,r3 < 0.5$$

14 $$\left\{ {\begin{array}{*{20}l} {z1 = X_{a1} + \sigma *MeanRule + randn*\frac{{X_{a2} - X_{a3} }}{{f\left( {X_{a2} } \right) - f\left( {X_{a3} } \right) + 1 }}} \hfill \\ {z2 = X_{best} + \sigma *MeanRule + randn*\frac{{X_{a1} - X_{a2} }}{{f\left( {X_{a1} } \right) - f\left( {X_{a2} } \right) + 1 }}} \hfill \\ \end{array} } \right.\,\,\,\,\,\,\,if\,\,\, r3 \ge 0.5$$

15 $$\sigma = \left( {2*r4 - 1} \right)*\alpha = \left( {2*r4 - 1} \right)*c*{\text{exp}}\left( { - d*\frac{g}{{g_{max} }}} \right)$$

where $c = 2, d = 4$ are two constant numbers and $r3$ and $r4$ are random numbers generated from the [0, 1] range.

##### Vector-combining phase

In this phase, INFO combines two vectors, $z1$ and $z2$, to improve diversity. If the random probability is less than 0.5, the new vector will be the same as the current vector. Otherwise, INFO will generate a new vector, as in Eq. (16).

16 $$X_{i}^{g + 1} = \left\{ {\begin{array}{*{20}l} {X_{i}^{g} ,} \hfill & {if\,\,\, r5 \ge 0.} \hfill \\ {\left\{ {\begin{array}{*{20}c} {z1 + \mu \left| {z1 - z2} \right|, r6 < 0.5} \\ {z2 + \mu \left| {z1 - z2} \right|, r6 \ge 0.5} \\ \end{array} } \right.,} \hfill & {if\,\,\, r5 < 0.5} \hfill \\ \end{array} } \right.$$

where $\mu = 0.05*randn$, $r5$ and $r6$ are random numbers generated from the (0, 1) range, and $X_{i}^{g + 1}$ is the new position of the current search agent.

##### Local search phase

**Figure 4:**
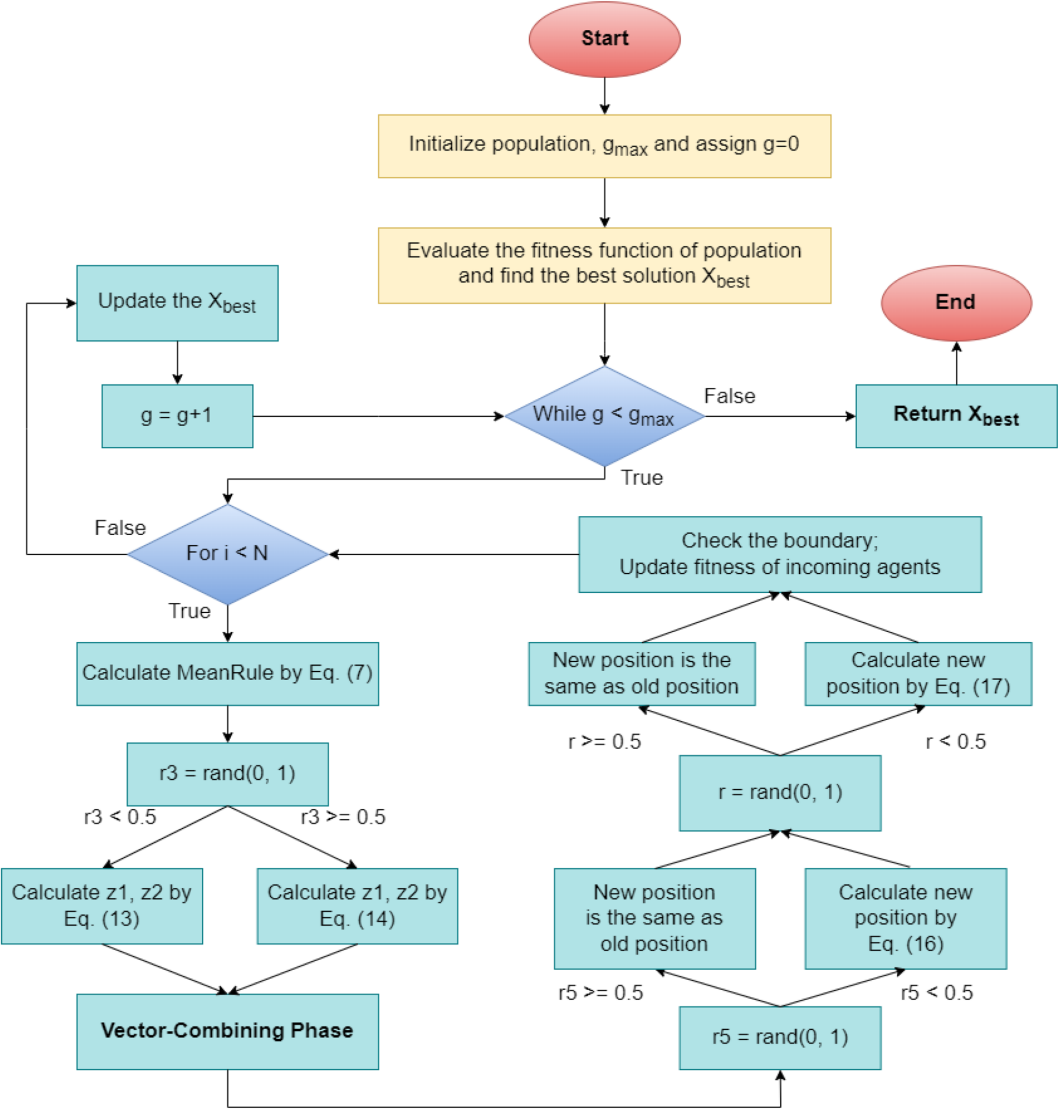
Weighted mean of vectors (INFO) optimization.

To further enhance the search performance, INFO utilizes a local search strategy around the global best solution and the solution derived from the mean-based rule. This phase is activated for the current agent only when the probability of a randomly generated number is smaller than 0.5. The agent skips this phase if the random probability is greater than or equal to 0.5. The new position of the current solution is presented in Eq. (17).

17 $$X_{i}^{g + 1} = \left\{ {\begin{array}{*{20}l} {X_{best} + randn*(MeanRule + randn*\left( {X_{best} - X_{a1} } \right), } \hfill & { if\,\,\,\,r7 < 0.5} \hfill \\ {X_{rnd} + randn*(MeanRule + randn*\left( {v_{1} *X_{best} - v_{2} *X_{rnd} } \right),} \hfill & { if\,\,\,\, r7 \ge 0.5} \hfill \\ \end{array} } \right.$$

18 $$X_{rnd} = \emptyset *X_{avg} + \left( {1 - \emptyset } \right)*\left( {\emptyset *X_{better} + \left( {1 - \emptyset } \right)*X_{best} } \right)$$

19 $$X_{avg} = \frac{{X_{a1} + X_{a2} + X_{a3} }}{3}$$

20 $$v1 = \left\{ {\begin{array}{*{20}l} {2*rand,} \hfill & { if\quad r8 > 0.5} \hfill \\ {1,} \hfill & { if\quad r8 \le 0.5} \hfill \\ \end{array} } \right.;\,\,\,v2 = \left\{ {\begin{array}{*{20}l} {2*rand,} \hfill & { if\quad r9 > 0.5} \hfill \\ {1,} \hfill & { if\quad r9 \le 0.5} \hfill \\ \end{array} } \right.$$

where $r7, r8$, $r9$ and $\emptyset$ are random numbers generated from the (0, 1) range. Finally, the flowchart of the INFO algorithm is presented in Fig. 4.

#### Runge Kutta optimization (RUN)

This section briefly overviews the Runge–Kutta optimization (RUN) algorithm^39^. The RUN algorithm is based on the Runge‒Kutta method, which is commonly used in numerical methods to solve ordinary differential equations. The RUN algorithm consists of three phases: the first phase is initialization (described above), the second phase involves a search procedure based on the Runge–Kutta theory, and the third phase is enhanced solution quality (ESQ). RUN has the benefit of being simple to execute and effective; therefore, it has been successfully applied in several fields, such as photovoltaic systems^47^, optimizing hydropower reservoirs^40^, and feature selection^48^. The subsequent subsection will briefly describe the RUN algorithm's main equations and fundamental concepts; more details are available at^39^.

##### Search mechanism-based Runge–Kutta theory

In this phase, the RUN algorithm uses a search mechanism (SM) to update the position of the current solution at each generation. It can be determined by Eq. (21).

21 $$X_{i}^{g + 1} = \left\{ {\begin{array}{*{20}l} {X_{c} + r4*SF*r5*X_{c} + SF*SM + \mu *X_{s1} ,} \hfill & { if\quad pr1 < 0.5} \hfill \\ {X_{m} + r4*SF*r5*X_{m} + SF*SM + \mu *X_{s2} , } \hfill & { if \quad pr1 \ge 0.5} \hfill \\ \end{array} } \right.$$

22 $$X_{s1} = randn*\left( {X_{m} - X_{c} } \right);\,\,\,\,X_{s2} = randn*\left( {X_{r1} - X_{r2} } \right)$$

23 $$X_{c} = \varphi X_{i} + \left( {1 - \varphi } \right)X_{r1} ;\,\,\,X_{m} = \varphi X_{best} + \left( {1 - \varphi } \right)X_{best}^{g}$$

24 $$SF = 2*\left( {0.5 - r6} \right)*f = 2*\left( {0.5 - r6} \right)*a*{\text{exp}}\left( { - b*r7*\frac{g}{{g_{max} }}} \right)$$

25 $$SM = \frac{1}{6}X_{rk} *\Delta x = \frac{1}{6}\left( {k_{1} + 2k_{2} + 2k_{3} + k_{4} } \right)*\Delta x$$

26 $$k_{1} = \frac{1}{2\Delta x}*\left( {rand*X_{wr}^{g} - u*X_{br}^{g} } \right)$$

where $$r4$$ is a random integer from the set {1,− 1} and $$r5$$ is a random number generated from the [0, 2] range. $$pr1$$, $$r6$$, $$r7$$, and $$\varphi$$ are random numbers generated from the (0, 1) range. $$a$$ and $$b$$ are two constant numbers. $$\mu = 0.5 + 0.1*randn$$ is a random number. $$X_{wr}^{g}$$ and $$X_{br}^{g}$$ are the worst random and best random solutions determined based on three random solutions ($$X_{r1} , X_{r2} , X_{r3} )$$ selected from the current population with condition $$r1 \ne r2 \ne r3 \ne i$$. The rule is defined as follows:

If the fitness of $$X_{i}$$ is better than the fitness of the best solution $$X_{bi}$$ selected from ($$X_{r1} , X_{r2} , X_{r3} )$$, then we have $$X_{wr}^{g} = X_{bi}$$ and $$X_{br}^{g} = X_{i}$$.

Otherwise, we have $X_{wr}^{g} = X_{i}$ and $X_{br}^{g} = X_{bi}$

27 $$\Delta x = 2*rand*\left| {rand*\left( {\left( {X_{br}^{g} - rand*X_{avg} } \right) + \gamma } \right)} \right|$$

28 $$\gamma = rand*(X_{i} - rand*\left( {X^{max} - X^{min} } \right))*{\text{exp}}\left( { - 4*\frac{g}{{g_{max} }}} \right)$$

29 $$k_{2} = \frac{1}{2\Delta x}\left[ {rand*\left( {X_{wr}^{g} + r8*k_{1} *\Delta x} \right) - \left( {u*X_{br}^{g} + r9*k_{1} *\Delta x} \right)} \right]$$

30 $$k_{3} = \frac{1}{2\Delta x}*\left[ {rand*\left( {X_{wr}^{g} + r8*\frac{{k_{2} }}{2}*\Delta x} \right) - \left( {u*X_{br}^{g} + r9*\frac{{k_{2} }}{2}*\Delta x} \right)} \right]$$

31 $$k_{4} = \frac{1}{2\Delta x}\left[ {rand*\left( {X_{wr}^{g} + r8*k_{3} *\Delta x} \right) - \left( {u*X_{br}^{g} + r9*k_{3} *\Delta x} \right)} \right]$$

32 $$X_{avg} = \frac{{X_{r1} + X_{r2} + X_{r3} }}{3}$$

where $r8$ and $r9$ are random numbers generated from the (0, 1) range.

##### Enhanced solution quality

**Figure 5:**
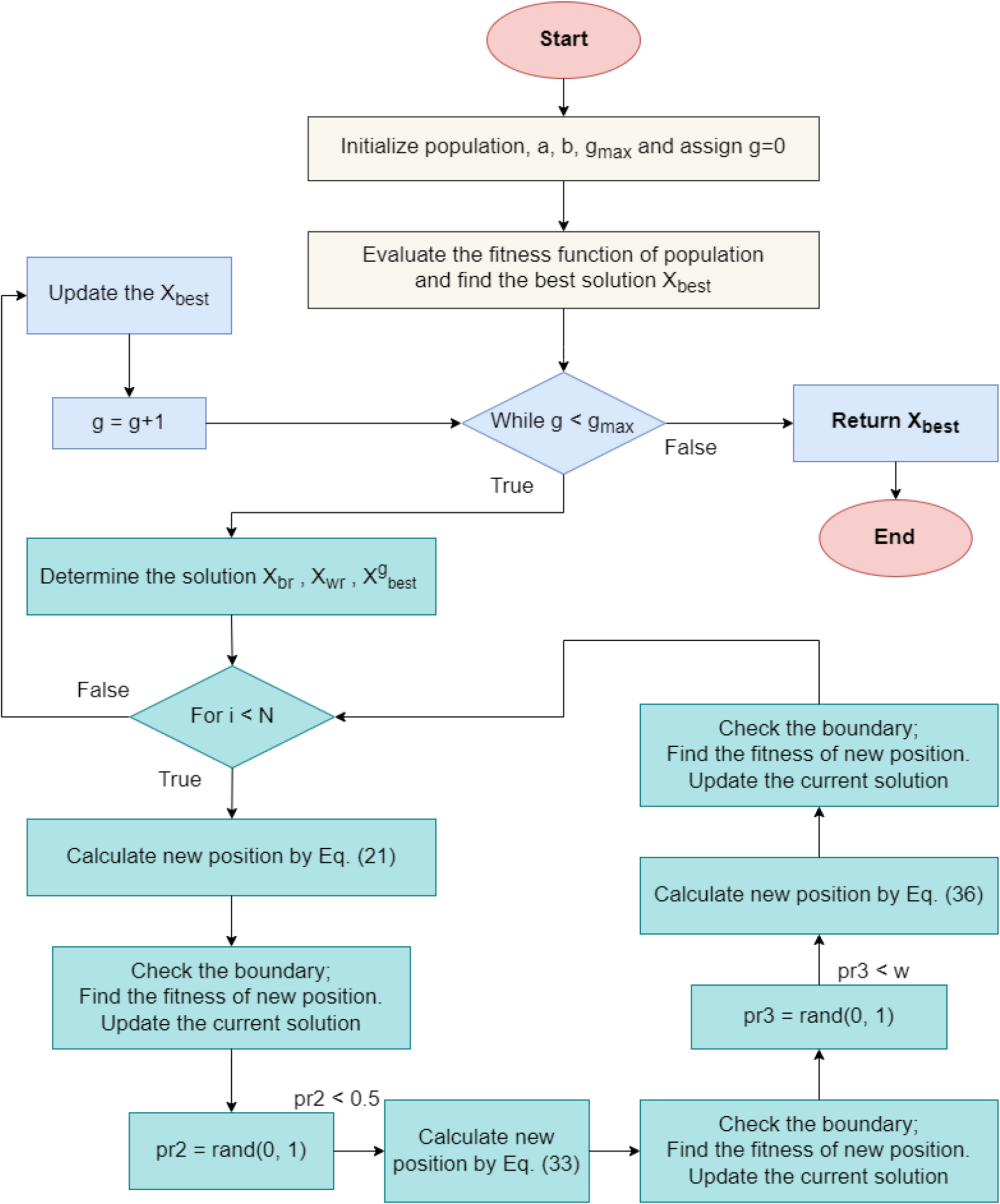
Runge–Kutta optimization (RUN) algorithm.

This phase is implemented in the RUN algorithm to increase the quality of the solutions and avoid local optima in each generation. Additionally, this phase is executed only when the probability of a random number is smaller than 0.5 ($$pr2 < 0.5)$$. The mathematical formulation can be described as Eq. 12

33 $$X_{i2} = \left\{ {\begin{array}{*{20}l} {X_{i1} + r10*w*\left| {X_{i1} - X_{avg} + randn} \right|,} \hfill & { if\quad w < 1.0} \hfill \\ {X_{i1} - X_{avg} + r10*w*\left| {u*X_{i1} - X_{avg} + randn} \right|,} \hfill & { if\quad w \ge 1.0} \hfill \\ \end{array} } \right.$$

34 $$w = rand\left( {0, 2} \right)*{\text{exp}}\left( { - c*\frac{g}{{g_{max} }}} \right)$$

35 $$X_{i1} = \beta *X_{avg} + \left( {1 - \beta } \right)*X_{best}$$

where $\beta$ is a random number generated from the (0, 1) range. $c = 5*rand$ is a random number. $r10$ is a random integer chosen from the set {-1, 0, 1}. The $w$ factor is used to control the exploitation and exploration process in this phase. For $w \ge 1$ (at early generations), the solution $X_{i2}$ tends to explore the search space. When $w < 1$ (at later generations), the solution $X_{i2}$ tends to exploit around the $X_{i1}$ position. To enhance the performance of the new solution, the RUN algorithm also utilizes the new operator when the newly generated solution X is not better than the current solution. It can be expressed using Eq. (36).

36 $$X_{i3} = X_{i2} - r11*X_{i2} + SF*\left( {r12* X_{rk} + v*X_{br}^{g} - X_{i2} } \right)$$

where $r11$ and $r12$ are random numbers in the (0, 1) range and $v = 2*rand$ is a random number. It is worth noting that the chance to generate $X_{i3}$ occurs when $pr3 < w$, where $pr3$ is a random number in the (0, 1) range. Finally, the flowchart of the RUN algorithm is shown in Fig. 5.

#### Extreme learning machine (ELM)

**Figure 6:**
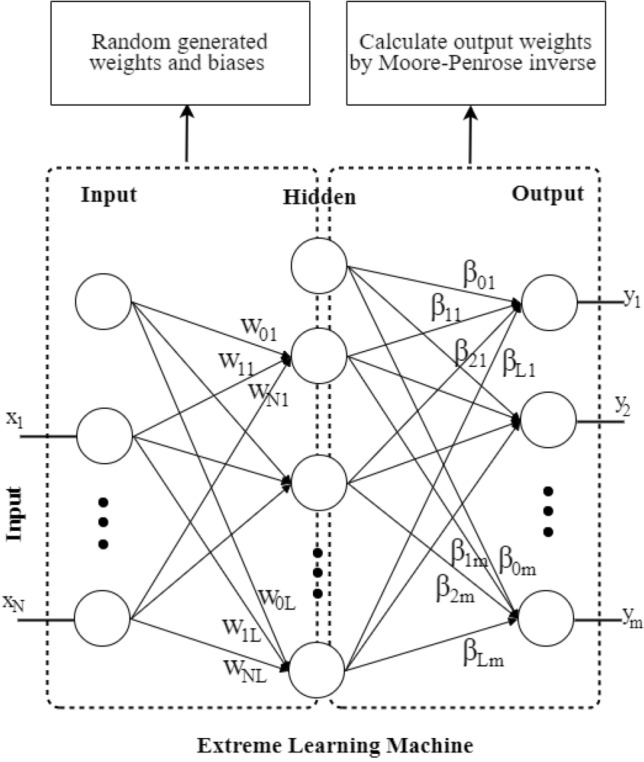
Extreme Learning Machine (ELM).

The extreme learning machine (ELM) algorithm was developed to improve the efficiency and speed of single-hidden-layer feedforward networks (SLFNs) without the need for tuning the hidden weights^23^. Unlike the MLP (ANN or FFNN), which requires an iterative optimization process to determine the hidden weights, the ELM randomly assigns them. The weights between the hidden and output layers are then calculated using the Moore–Penrose inverse method (Fig. 6). ELM is an “extreme” approach because the hidden weights are adjusted only once during training and are randomly assigned^33^. This enables the ELM to be trained much faster than traditional neural networks and handle large amounts of data with high-dimensional input spaces. ELM has been successfully applied in a variety of fields, including environmental sciences^49^, time-series forecasting^50^, and agriculture^51^.

The most commonly used structure of the ELM is based on an MLP network^52^ with an input layer, a single hidden layer and an output layer. Given a set of training pairs {$\left( {x_{i} , y_{i} } \right) \in R^{N}$ x $R^{m}$}, where $x_{i}$ is the input vector with N dimensions and $y_{i}$ is the m-dimensional output vector, the output of neuron $j^{th}$ of the ELM with L hidden neurons can be expressed as follows:

37 $$\hat{y}_{k} = \mathop \sum \limits_{j = 1}^{L} \beta_{kj} .G_{j} \left( {\mathop \sum \limits_{i = 1}^{N} W_{ji} .x_{i} + b_{j} } \right)$$

where $G()$ is the activation function and $W_{ji}$ and $b_{j}$ are the weights and biases between the input and hidden layers, respectively. $x_{i}$ is the $i^{th}$ input node, and $\beta_{kj}$ is the between hidden and output layers. The training process of ELM is executed as follows:

- Randomly generated the $W_{ji}$ and $b_{j}$
- Calculate the $\beta_{kj}$ by solving least squares problem $Y = \beta H$, where $Y$ is the true output (observation values), $H$ is the output matrix of the hidden layer. $\beta$ can be calculated using Moore–Penrose inverse which is $\beta = H^{ + } Y = \left( {H^{T} H} \right)^{ - 1} H^{T} Y$

#### Proposed models

##### Training process of hybrid-based ELM models

Although the ELM employs Moore–Penrose to find the global optimal point for the least squares problem, the matrix H contains random components of the hidden weights. As these weights are generated only once during the training process and are not updated, the use of Moore–Penrose will only locate nonglobal optimal points if the initially generated weights are not globally optimal^33^. This is the main drawback of the ELM model. Therefore, in this paper, metaheuristic algorithms are proposed to update and find better sets for hidden weights of the ELM network, followed by using Moore–Penrose to calculate the output weights.

Before applying the metaheuristic algorithms, it is essential to define two critical components: the representation of a solution for the optimization problem and the fitness function. These components are addressed for the proposed hybrid-based ELM model (where hybrid-based refers to the proposed algorithms such as PSS, INFO, RUN, and other algorithms that will be compared in the subsequent sections of this study) as follows:

In the proposed hybrid-based ELM model, let $s_{i} , s_{h}$ and $s_{o}$ be the sizes of the input, hidden and output layers, respectively. Vectors $\vec{W}$ and $\vec{b}$ are the weights and biases of the input and hidden layers, respectively, in the ELM network. A solution is a real vector $\vec{S}$ (solution), which includes two components: input weights and hidden biases, represented as follows. All values are generated within the range [− 1, 1]:

$$\vec{S} = \left\{ {\vec{W},\vec{b}} \right\} = \left\{ {W_{1,1} \ldots W_{1,L,} W_{N,1} \ldots W_{NL,} ,b_{1} \ldots b_{L} } \right\}$$

All values are generated within the range [− 1, 1]. This vector $\vec{S}$ encapsulates the necessary parameters for the ELM model, which will be optimized using the proposed metaheuristic algorithms.

In metaheuristic algorithms, the fitness function evaluates the quality of a given solution. In this study, the average root mean square error ($\overline{RMSE}$) is selected as the fitness function, a common choice in hybrid-based ELM models. The $\overline{RMSE}$ is calculated by averaging the RMSE over the entire training dataset, as shown in Eq. (39). In which, Eq. (38) defines the RMSE based on the difference between the ground-truth value and the ELM output.

38 $$RMSE = \sqrt {\mathop \sum \limits_{i = 1}^{m} \left( {{\hat{\text{y}}}_{i} - y_{i} } \right)^{2} }$$

39 $$\overline{RMSE} = \frac{1}{{s_{ts} }}\mathop \sum \limits_{j = 1}^{{s_{ts} }} RMSE_{j} = \frac{1}{{s_{ts} }}\mathop \sum \limits_{j = 1}^{{s_{ts} }} \sqrt {\mathop \sum \limits_{i = 1}^{m} \left( {{\hat{\text{y}}}_{i} - y_{i} } \right)^{2} }$$

where $s_{ts}$ denotes the size of the training set, ${\hat{\text{y}}}_{i}$ represents the output of the ELM network (as per Eq. (37)), and $y_{i}$ represents the corresponding ground truth value.

In this manner, the training process of the ELM network is formulated as an optimization problem aimed at minimizing the RMSE value. Similarly, the hybrid-ELM model can minimize the F($\vec{S}$) = $\overline{RMSE}$ function. Specifically, the procedure of the proposed hybrid-based ELM models can be described as follows: The proposed algorithm initializes a population of N solutions randomly. Each solution represents a candidate ELM network, as described above. Then, the fitness for each solution is calculated through the following steps:

- Convert the solution back into input weights and hidden biases, then assign them to the ELM network.
- Compute the output weights of the ELM using the Pseudo-Inverse Method based on the training dataset.
- Evaluate the $\overline{RMSE}$ value based on the validation part of the dataset.

After calculating the $\overline{RMSE}$ value, it is returned to the fitness function of the metaheuristic algorithms. The algorithms then perform the operation steps described in the earlier sections to evolve the population. This process repeats iteratively until the stopping condition of the algorithm is met. At this point, the solution with the best performance (i.e., the lowest $\overline{RMSE}$) is returned to the ELM network. Consequently, we obtain the optimal and most effective ELM model.

##### Proposed hybrid-based ELM

**Figure 7:**
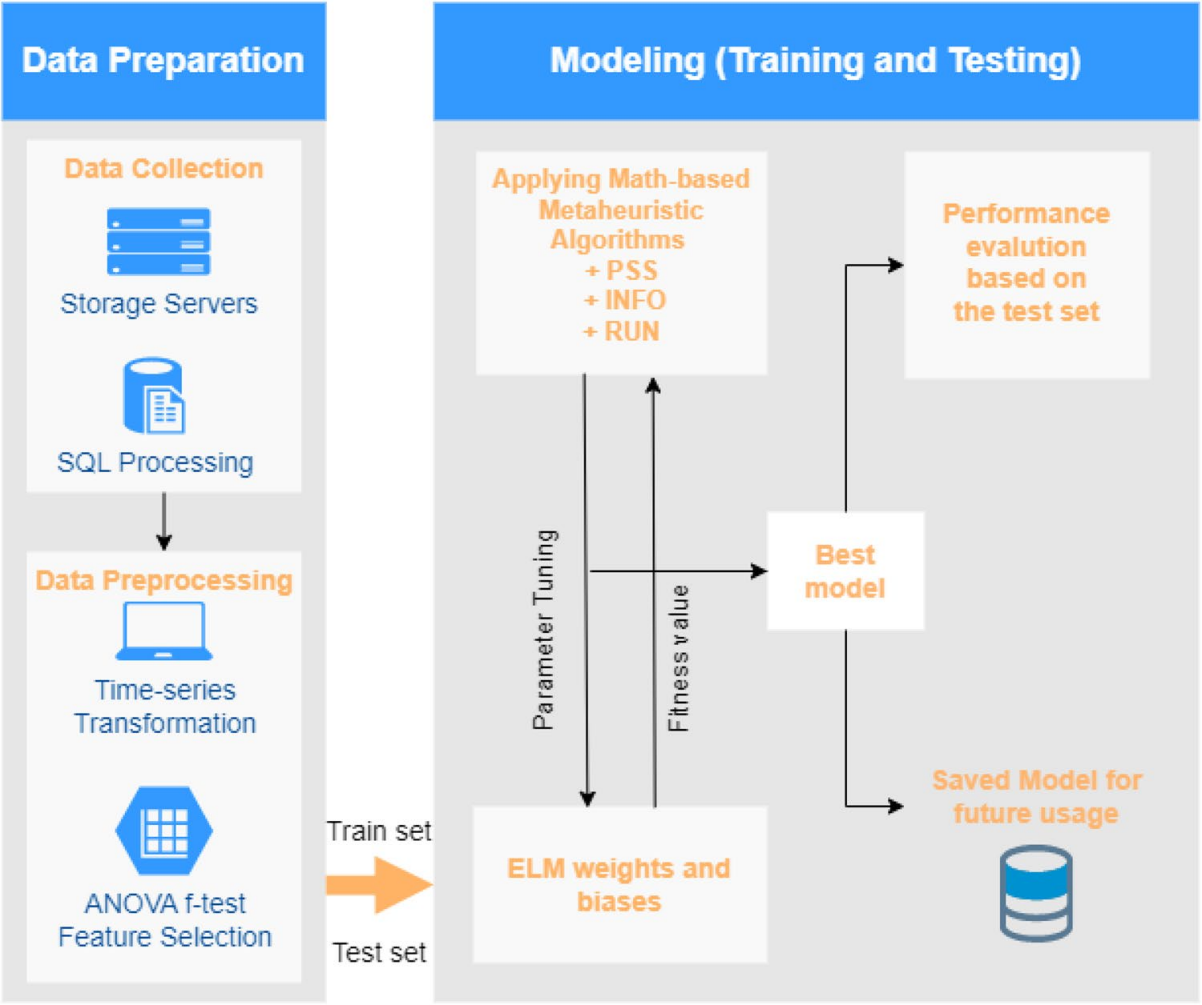
Metaheuristic-based ELM Model.

This section provides a detailed description of our proposed model, illustrated in Fig. 7. Our model consists of two main components: Data Preparation and Modeling (Training and Testing).

The Data Preparation component aims to collect and prepare data for the model. During the data collection phase, the Data Collection component stores data on servers and retrieves it in CSV format using SQL queries. These CSV files are then passed through the Data Preprocessing component for cleaning and preparation for the model. To use time-series data for machine learning models (i.e. neural network), the first step is to transform it to a supervised learning format using the sliding windows method. The sliding window's width represents the time series' lag values. Next, we implement the ANOVA F-test^53^ to determine the model's appropriate input variables (best lagged features). This approach is widely employed in hydrology-related domains^29,54^. According to the findings of the ANOVA F-test, six lagged times are chosen as input features for one-step ahead river streamflow modeling as shown in Table 2.

**Table 2:** The input features and output for tested models.

Input variables	Output variable
Q(t−1), Q(t−11), Q(t−12), Q(t−13), Q(t−23), Q(t−24)	Q(t)

where Q(t) is the target river streamflow step "one month ahead" and Q(t − 1),…, Q(t − 24) are the lag times feature up to 24 months previously. Here, six features with the highest ANOVA F-test value are selected as input variables for proposed models. And then, the data were split into two parts: training and testing.

The Modeling component is responsible for training the model, storing the best model, and evaluating the results. First, the model is built using the training set by encoding the weights and biases of the ELM network into solutions. Then, the proposed metaheuristic algorithms are used to optimize these solutions. After the training and parameter tuning processes are completed, the best model is saved for future use and evaluated on the test set for performance analysis. During the building phase, the root mean square error (RMSE) function is used as the fitness function to evaluate the model's performance. Six famous performance metrics are used during the evaluation phase, which are described in detail in the following section.

## Experiments

### Compared models and parameter settings

To assess the ability of the three proposed optimizers for modeling river streamflow data, 17 other well-known metaheuristic algorithms from different groups of nature-inspired algorithms were selected for comparison. All 20 metaheuristic algorithms are used to optimize the weights for the ELM network. Note that the implemented codes for all metaheuristic algorithms are available at^34^.

To ensure objectivity in the comparison process, the parameters for the metaheuristic algorithms were taken from their original paper and then modified through the parameter tuning process to select the best set of parameter. Moreover, to ensure fairness, all algorithms will have a population size of 50 and a maximum number of function evaluations of 50,000. This study does not use a maximum number of iterations because many algorithms update positions and fitness multiple times within a single iteration. Therefore, the maximum number of function evaluations is necessary to ensure fairness. Table 3 shows all 20 algorithms used in the experiments and their best parameters (N/A is used to indicate that no additional parameter is required). In addition to the parameters for the above algorithms, other parameters for the model and data were set as follows:

- The training and testing data split ratios are 80% and 20%, respectively, for all the models.
- The ELM network has three layers: 1 input layer, 1 hidden layer, and 1 output layer.
- The number of input nodes in the ELM network is $s_{i}$ (selected based on the ANOVA feature selection method mentioned above). The number of hidden nodes in the ELM network is 2*$s_{i}$ + 1, following the formula from previous papers. The number of output nodes is 1, and the activation function used is the ELU.

**Table 3:** The tested models and hyperparameter settings.

Group	Model	Name	Parameters
Evolutionary	GA-ELM	Genetic Algorithm^55^	Crossover probability $p_{c} = 0.85$ and the mutation probability $p_{m} = 0.05$
	CRO-ELM	Coral Reefs Optimization^56^	$p_{o} = 0.85$, $F_{b} = 0.9$, $F_{a} = 0.1$, $F_{d} = 0.1$, $P_{d} = 0.1$, $GCR = 0.1$, gamma $\gamma_{min} = 0.02$, gamma $\delta_{max} = 0.02$
Swarm	AGTO-ELM	Artificial Gorilla Troops Optimization^57^	$p = 0.05$, $w = 0.8$, updating coefficient $beta = 3.0$
	DMOA-ELM	Dwarf Mongoose Optimization Algorithm^58^	N/A
	HGS-ELM	Hunger Games Search^59^	$pup = 0.03$, $LH = 1000$
	WOA-ELM	Whale Optimization Algorithm^60^	N/A
Physics	NRO-ELM	Nuclear Reaction Optimization^61^	N/A
	HGSO-ELM	Henry Gas Solubility Optimization^62^	Number of clusters $n_{clusters} = 2$
	ASO-ELM	Atom Search Optimization^63^	Depth weight $\alpha = 10$, multiplier $\beta = 0.2$
Human	GSKA-ELM	Gaining Sharing Knowledge-based Algorithm^64^	$p_{b} = 0.1$, $k_{f} = 0.5$, knowledge ratio $k_{r} = 0.9$,$k_{g} = 5$
	LCO-ELM	Life Choice-based Optimization^65^	Step size $r_{1} = 2.35$
Biology	SMA-ELM	Slime Mould Algorithm^66^	Probability threshold $p_{t} = 0.03$
	SOA-ELM	Seagull Optimization Algorithm^67^	Frequency of employing $fc = 2$
	TSA-ELM	Tunicate Swarm Algorithm^68^	N/A
System	AEO-ELM	Artificial Ecosystem-based Optimization^54^	N/A
Music	HS-ELM	Harmony Search^69^	Consideration rate $c_{r} = 0.95$, pitch adjustment rate $pa_{r} = 0.05$
Math	GBO-ELM	Gradient-Based Optimizer^70^	$p_{r} = 0.5$, $\beta_{min} = 0.2$,$\beta_{max} = 1.2$
	PSS-ELM	Pareto-like Sequential Sampling	Acceptance rate $ar = 0.9$, $sampling = LHS$ (Latin-Hypercube)
	INFO-ELM	weighted meaN oF vectOrs	N/A
	RUN-ELM	RUNge Kutta optimizer	$a = 20, b = 12$

### Performance metrics

As stated above, the RMSE metric was utilized as the fitness function for the metaheuristic algorithms during the model training process. However, during the testing phase, various metrics are employed, as listed in Table 4. All of the source code for the performance metrics is available at^71^.

**Table 4:** Descriptions of the performance metrics.

Metrics	Equation	Characteristics
Mean absolute error	$MAE = \frac{{\mathop \sum \nolimits_{i = 1}^{N} \left| {\hat{y} - y} \right|}}{N}$	Smaller is better (best = 0), range = (− inf, + inf)
Root mean square error	$RMSE = \sqrt {\frac{{\mathop \sum \nolimits_{i = 1}^{N} \left( {\hat{y} - y} \right)^{2} }}{N}}$	Smaller is better (best = 0), range = (− inf, + inf)
Pearson’s correlation coefficient		Greater is better (best = 1), range = (− inf, 1]
Mean absolute percentage error	$MAPE = \frac{1}{N}*\mathop \sum \limits_{i = 1}^{N} \left| {\frac{{y - \hat{y}}}{y}} \right|$	Smaller is better (best = 0), range = [0, + inf)
Nash–sutcliffe efficiency	$NSE = 1 - { }\frac{{\mathop \sum \nolimits_{1}^{N} \left( {y_{i} - \hat{y}_{i} } \right)^{2} }}{{\mathop \sum \nolimits_{i = 1}^{N} \left( {y_{i} - mean\left( Y \right)} \right)^{2} }}$	Greater is better (best = 1), range = (− inf, 1]
Kling-Gupta efficiency	$$KGE = 1 - \sqrt {\left( {R - 1} \right)^{2} + \left( {\beta - 1} \right)^{2} + \left( {\gamma - 1 } \right)^{2} }$$ R is Pearson’s Correlation Coefficient, CV is coefficient of variation, μ is mean, σ is standard deviation $$\beta = {\raise0.7ex\hbox{${\mu_{{\hat{y}}} }$} \!\mathord{\left/ {\vphantom {{\mu_{{\hat{y}}} } {\mu_{y} }}}\right.\kern-0pt} \!\lower0.7ex\hbox{${\mu_{y} }$}}$$; $$\gamma = {\raise0.7ex\hbox{${CV_{{\hat{y}}} }$} \!\mathord{\left/ {\vphantom {{CV_{{\hat{y}}} } {CV_{y} }}}\right.\kern-0pt} \!\lower0.7ex\hbox{${CV_{y} }$}} = \frac{{\sigma_{{\hat{y}}} \mu_{y} }}{{\mu_{{\hat{y}}} \sigma_{y} }}$$	Greater is better (best = 1), range = (− inf, 1]

## Results and discussion

This section presents a comprehensive set of experimental results concerning 20 metaheuristic algorithms used for optimizing the ELM network. These algorithms belong to various branches of nature-inspired algorithms. Notably, three recent math-based metaheuristic algorithms, namely, PSS, INFO, and RUN, which are included at the end of the figures and tables, were used. Moreover, the tables highlight these algorithms in bold for ease of identification. As metaheuristic algorithms are stochastic (randomly based), each model was executed 10 times, and the mean and standard deviation (std) were recorded.

### Statistical results

Table 5 encapsulates the mean performance values derived from 20 models following 10 iterative trials conducted on both the training and testing datasets. The evaluation of these models relied on six essential performance metrics: mean absolute error (MAE), root mean square error (RMSE), correlation coefficient (R), mean absolute percentage error (MAPE), Nash–Sutcliffe efficiency (NSE), and Kling-Gupta efficiency (KGE). By analyzing the training dataset, it becomes evident that our proposed RUN-ELM model shows superior performance across all the considered metrics. This model exhibits the lowest values for the MAE, RMSE, and MAPE, underscoring its precision in capturing the discrepancies between the predicted and observed streamflow values. Moreover, the RUN-ELM yields the highest correlation coefficient (R), Nash–Sutcliffe efficiency (NSE), and Kling-Gupta efficiency (KGE) values. These results illuminate its capacity not only for accurate prediction but also for maintaining consistency and robustness across varying streamflow scenarios. Conversely, several models, namely, HGSO-ELM, ASO-ELM, GSKA-ELM, LCO-ELM, SMA-ELM, SOA-ELM, TSA-ELM, and AEO-ELM, demonstrate comparatively inferior performance metrics on the training dataset.

**Table 5:** The mean results of all compared models after 10 trials on various performance metrics.

Model	Train						Test					
	MAE	RMSE	R	MAPE	NSE	KGE	MAE	RMSE	R	MAPE	NSE	KGE
GA-ELM	0.9465	1.5306	0.9777	0.1460	0.9559	0.9684	1.1986	2.0963	**0.9376**	0.1572	0.8604	0.9173
CRO-ELM	0.9011	1.4510	0.9800	0.1401	0.9603	0.9717	1.2243	2.2149	0.9315	0.1530	0.8440	0.9128
AGTO-ELM	0.9276	1.4799	0.9792	0.1463	0.9587	0.9706	1.2169	2.1438	0.9336	0.1577	0.8539	0.9171
DMOA-ELM	0.9283	1.4940	0.9788	0.1454	0.9580	0.9699	1.1980	2.1245	0.9372	0.1556	0.8566	0.9124
HGS-ELM	0.9958	1.6318	0.9746	0.1526	0.9498	0.9641	**1.1875**	2.0687	0.9344	0.1607	0.8640	0.9094
WOA-ELM	0.9665	1.5680	0.9765	0.1490	0.9537	0.9670	1.2236	2.1547	0.9316	0.1611	0.8524	0.9123
NRO-ELM	0.9347	1.4978	0.9786	0.1451	0.9577	0.9699	1.2185	2.1344	0.9343	0.1583	0.8552	**0.9175**
HGSO-ELM	1.0826	1.7630	0.9703	0.1649	0.9414	0.9579	1.2493	2.1752	0.9276	0.1626	0.8497	0.9147
ASO-ELM	1.1138	1.8106	0.9686	0.1690	0.9382	0.9566	1.3018	2.2657	0.9261	0.1706	0.8367	0.9043
GSKA-ELM	1.0362	1.6427	0.9742	0.1645	0.9492	0.9639	1.2828	2.1456	0.9335	0.1795	0.8537	0.9012
LCO-ELM	1.0304	1.6920	0.9727	0.1560	0.9460	0.9614	1.1942	2.0910	0.9324	0.1602	0.8611	0.9081
SMA-ELM	1.0414	1.6961	0.9725	0.1615	0.9458	0.9611	1.2451	2.1168	0.9299	0.1739	0.8576	0.8937
SOA-ELM	1.0613	1.7273	0.9715	0.1622	0.9438	0.9594	1.2258	2.1223	0.9301	0.1675	0.8569	0.9015
TSA-ELM	1.0133	1.6148	0.9751	0.1618	0.9508	0.9649	1.2290	2.0979	0.9362	0.1661	0.8601	0.9114
AEO-ELM	1.0032	1.6425	0.9743	0.1531	0.9492	0.9635	1.2246	2.1470	0.9298	0.1607	0.8535	0.9133
HS-ELM	0.9150	1.4777	0.9792	0.1404	0.9588	0.9706	1.2520	2.2857	0.9265	0.1573	0.8338	0.9069
GBO-ELM	0.9127	1.4670	0.9795	0.1416	0.9595	0.9710	1.2094	2.1436	0.9356	0.1553	0.8540	0.9131
**PSS-ELM**	0.9965	1.5885	0.9759	0.1616	0.9525	0.9658	1.2127	**2.0667**	0.9374	0.1660	**0.8642**	0.9148
**INFO-ELM**	0.8975	1.4464	0.9801	0.1371	0.9606	0.9718	1.2145	2.1937	0.9321	**0.1521**	0.8471	0.9113
**RUN-ELM**	**0.8891**	**1.4359**	**0.9804**	**0.1349**	**0.9612**	**0.9723**	1.2105	2.1586	0.9360	0.1528	0.8518	0.9124

Significant values are in bold.

These models are characterized by elevated values of MAE, RMSE, and MAPE, indicating a larger magnitude of prediction errors. Furthermore, they exhibit lower correlation coefficient (R), Nash–Sutcliffe efficiency (NSE), and Kling-Gupta efficiency (KGE) values, suggesting diminished accuracy, consistency, and model efficiency. The dominance of the RUN-ELM in the training dataset highlights its ability to capture the intricate patterns and dynamics of streamflow, leading to more accurate and reliable predictions. The robust performance of this model across multiple evaluation metrics signifies its potential for practical application in streamflow forecasting systems, particularly in scenarios where precision and stability are paramount.

These findings offer valuable insights into the effectiveness of the proposed RUN-ELM model, emphasizing its role as a promising tool for optimizing water resource management strategies and mitigating potential risks associated with streamflow variations. In conclusion, the comprehensive evaluation of these models underscores the superiority of the RUN-ELM in streamflow forecasting applications, providing the way for further research and improvement of math-inspired metaheuristic approaches in hydrological modeling and water resource management environments.

Following an examine the model assessment on the test dataset, we can see that our proposed PSS-ELM model emerges as the top-performing model across two crucial performance metrics: the root mean square error (RMSE) and the Nash–Sutcliffe efficiency (NSE). The PSS-ELM model achieves an RMSE of 2.0667, indicating that it can forecast streamflow values with minimum errors. Additionally, the high NSE value of 0.8642 further underscores the model’s ability to capture the variability of streamflow patterns with a notable degree of accuracy.

Intriguingly, the results reveal that the GA-ELM model exhibits the highest correlation coefficient (R) among all the models, reaching 0.9376. This signifies the robustness of the GA-ELM in establishing strong linear relationships between the predicted and observed streamflow values. Notably, the PSS-ELM model closely follows, with the second-highest R value of 0.9374, reinforcing its efficacy in capturing the underlying patterns within the streamflow dataset. Furthermore, when considering the Kling-Gupta efficiency (KGE) metric, the differences in performance between the best-performing model, NRO-ELM, and PSS-ELM model are relatively small (0.9175 and 0.9148, respectively). This implies that the PSS-ELM model demonstrates remarkable efficiency in replicating the observed variability of the target variable within the test dataset.

It can be seen from Table 5 that ASO-ELM has the lowest performance across multiple metrics. It exhibits relatively higher MAE, RMSE, MAPE, and lower R, NSE, and KGE values compared to many other models listed. On the other hand, the PSS-ELM model performs competitively in terms of most metrics. It achieves relatively low MAE and RMSE values, high R value, and a significantly lower MAPE compared to many other models, indicating its superior accuracy in streamflow prediction. Additionally, its NSE and KGE values are also relatively high, indicating good agreement with observed data.

The superior performance of the PSS-ELM model across the RMSE and NSE metrics signifies its ability to minimize prediction errors and accurately capture overall streamflow dynamics. The high correlation coefficient (R) values attained by both the PSS-ELM and GA-ELM models reflect their capacity to establish robust linear relationships, which is crucial for precise streamflow predictions. Moreover, the small disparity in KGE values between NRO-ELM and PSS-ELM further highlights the commendable performance of the PSS-ELM model in replicating the observed variability and overall characteristics of streamflow in the test dataset. This suggests that the PSS-ELM model exhibits a strong ability to adapt to varying streamflow conditions, demonstrating its reliability and effectiveness in streamflow forecasting tasks.

In summary, the results underscore the superior performance of the PSS-ELM model in capturing the intricate dynamics of streamflow, as evidenced by its top rankings in terms of the RMSE and NSE metrics on the test dataset. The model's ability to closely follow the correlation coefficient (R) with the GA-ELM model and its competitive KGE value further solidify its position as a robust and accurate tool for streamflow forecasting. These findings illuminate the potential of the PSS-ELM model as a valuable asset in water resource management, offering reliable insights into streamflow behavior and aiding decision-making processes for sustainable water allocation and mitigation strategies.

### Model stability

Model stability, as indicated by low standard deviation (std) values in forecast performance indicators, is critical in real-world river flow prediction applications. Low std values suggest that the model's predictions are consistently near to actual values throughout various scenarios, which is important for stakeholders who rely on these predictions for decision-making because it decreases the likelihood of unexpected variances and errors. In real-world applications, particularly hydrology, accurate predictions are critical for managing water resources, and guaranteeing the effective operation of hydropower facilities. A model with low standard deviations is more reliable, giving confidence in operational decisions based on its forecasts. Furthermore, a low standard deviation in model performance indicates that the model retains its accuracy in a variety of hydrological situations, including changing rainfall patterns, seasonal variations, and extreme events. This resilience is especially crucial for managing and planning for climate variability and change. Moreover, accurate and steady estimates help optimize the allocation and use of water resources, minimizing waste and ensuring that water supplies meet demand efficiently.

To further investigate the stability of the models, an examination of their standard deviation (std) values following 10 independent runs offers valuable insights into the consistency of their performance during training and prediction phases. Table 6 provides a detailed overview of the std values across six key performance metrics, shedding light on the variability in model outcomes. The std values serve as a measure of dispersion, indicating how much the performance metrics deviate from the mean across multiple runs. Lower std values suggest more stable and consistent performance, whereas higher values indicate greater variability in the results. Upon analyzing the std values, notable observations emerge regarding the stability of the models on both the training and testing datasets. The SMA-ELM model, for instance, demonstrates remarkable stability on the training dataset, boasting the four best std values for metrics such as the root mean square error (RMSE), Pearson's correlation coefficient (R), Nash–Sutcliffe efficiency (NSE), and Kling-Gupta efficiency (KGE). This suggests that the SMA-ELM model consistently produces reliable results across different training runs.

**Table 6:** The standard deviation (std) results of all compared models after 10 trials on various performance metrics.

Model	Train						Test					
	MAE	RMSE	R	MAPE	NSE	KGE	MAE	RMSE	R	MAPE	NSE	KGE
GA-ELM	0.0155	0.0224	0.0007	0.0057	0.0013	0.0009	0.0357	0.0718	0.0045	0.0063	0.0095	0.0082
CRO-ELM	0.0193	0.0346	0.0010	0.0050	0.0019	0.0014	0.0473	0.0900	0.0055	0.0073	0.0126	0.0082
AGTO-ELM	0.0200	0.0196	0.0006	0.0065	0.0011	0.0007	0.0615	0.0784	0.0043	0.0115	0.0108	0.0083
DMOA-ELM	0.0143	0.0107	0.0003	0.0079	0.0006	0.0006	**0.0264**	**0.0572**	0.0040	0.0057	**0.0078**	0.0074
HGS-ELM	0.0218	0.0265	0.0008	0.0104	0.0016	0.0012	0.0369	0.0657	0.0041	0.0109	0.0086	0.0123
WOA-ELM	0.0213	0.0447	0.0013	0.0055	0.0026	0.0018	0.0605	0.0944	0.0068	0.0120	0.0127	0.0106
NRO-ELM	0.0140	0.0256	0.0007	0.0044	0.0014	0.0011	0.0355	0.0733	0.0055	0.0066	0.0101	0.0078
HGSO-ELM	0.0218	0.0178	0.0006	0.0090	0.0012	0.0014	0.0680	0.0709	0.0042	0.0136	0.0098	0.0107
ASO-ELM	0.0229	0.0233	0.0008	0.0087	0.0016	0.0024	0.0816	0.1040	0.0057	0.0147	0.0150	0.0144
GSKA-ELM	0.0149	0.0193	0.0006	0.0054	0.0012	0.0009	0.0536	0.0719	0.0043	0.0182	0.0098	0.0142
LCO-ELM	0.0249	0.0352	0.0012	0.0064	0.0022	0.0015	0.0406	0.0719	0.0053	0.0109	0.0097	0.0078
SMA-ELM	0.0221	**0.0063**	**0.0002**	0.0114	**0.0004**	**0.0003**	0.0476	0.0673	0.0036	0.0135	0.0089	0.0135
SOA-ELM	0.0110	0.0229	0.0008	0.0048	0.0015	0.0009	0.0365	0.0658	0.0040	0.0099	0.0090	0.0120
TSA-ELM	0.0251	0.0419	0.0013	0.0083	0.0025	0.0018	0.0553	0.0712	0.0041	0.0098	0.0096	0.0088
AEO-ELM	0.0199	0.0259	0.0008	0.0064	0.0016	0.0013	0.0668	0.0840	**0.0032**	0.0156	0.0116	0.0108
HS-ELM	0.0175	0.0392	0.0011	0.0039	0.0022	0.0016	0.0548	0.1139	0.0068	0.0076	0.0170	0.0107
GBO-ELM	**0.0069**	0.0115	0.0003	0.0030	0.0006	0.0005	0.0434	0.0707	0.0043	0.0063	0.0095	**0.0057**
PSS-ELM	0.0216	0.0148	0.0005	0.0090	0.0009	0.0010	0.0627	0.0754	0.0045	0.0181	0.0101	0.0108
INFO-ELM	0.0199	0.0401	0.0011	0.0037	0.0022	0.0016	0.0285	0.0659	0.0048	0.0063	0.0092	0.0070
RUN-ELM	0.0096	0.0154	0.0004	**0.0027**	0.0008	0.0006	0.0433	0.1010	0.0038	**0.0052**	0.0140	0.0112

Significant values are in bold.

In contrast, the DMOA model emerges as the most stable on the test dataset, showing the three best std values for the metrics of mean absolute error (MAE), RMSE, and NSE. This indicates that the DMOA model maintains a consistent level of performance when applied to unseen data, reinforcing its reliability in real-world predictive scenarios. However, it is essential to note that stability alone does not guarantee effective predictive performance. Despite their stability, both the SMA-ELM and DMOA models exhibit suboptimal predictive capabilities, as highlighted in Table 6. This emphasizes the need for a balanced assessment that considers both stability and predictive accuracy in model selection. Our proposed RUN-ELM model emerges as a promising candidate, demonstrating the best std value for the mean absolute percentage error (MAPE) metric on both the training and testing datasets. This indicates that the RUN-ELM model maintains consistent and reliable performance across multiple runs, particularly in terms of percentage error.

Additionally, the proposed INFO-ELM model shows commendable std values on the test dataset, positioning it favorably among the compared models. For instance, while the DMOA-ELM model exhibits the best std value of 0.0264 for the MAE, the INFO-ELM model closely follows, with a value of 0.0285, ranking second. Similarly, for the KGE metric, the best model records a std value of 0.0057 (GBO-ELM), with INFO-ELM securing a value of 0.007, ranking second. In summary, the proposed INFO-ELM and RUN-ELM models demonstrate notable stability across multiple performance metrics compared to the other models under consideration. These results highlight the significance of assessing mean performance as well as model stability through standard values, offering both scholars and practitioners important information in selecting solid and consistent models for particular applications.

**Figure 8:**
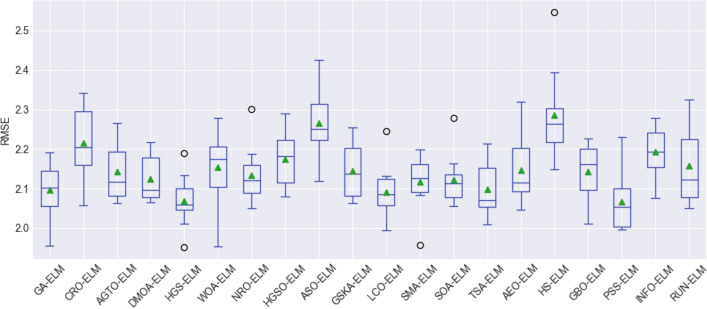
The RMSE results of all compared models after 10 trials (box plot).

**Figure 9:**
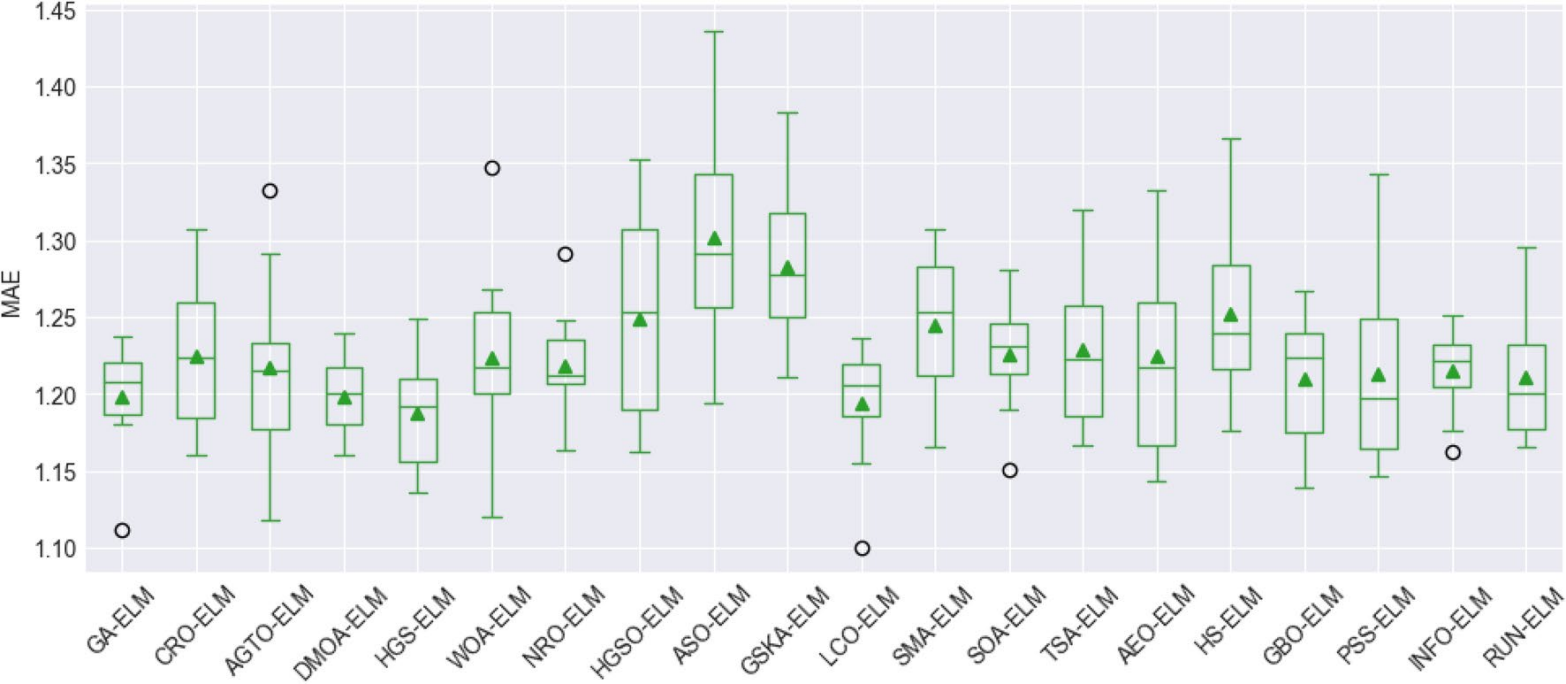
The MAEs of all compared models after 10 trials (box plot).

**Figure 10:**
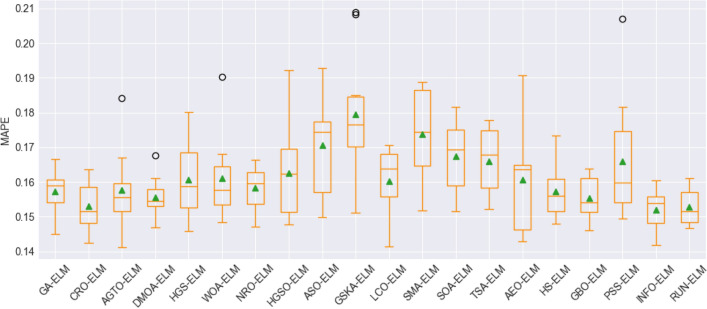
MAPE results of all compared models after 10 trials (box plot).

**Figure 11:**
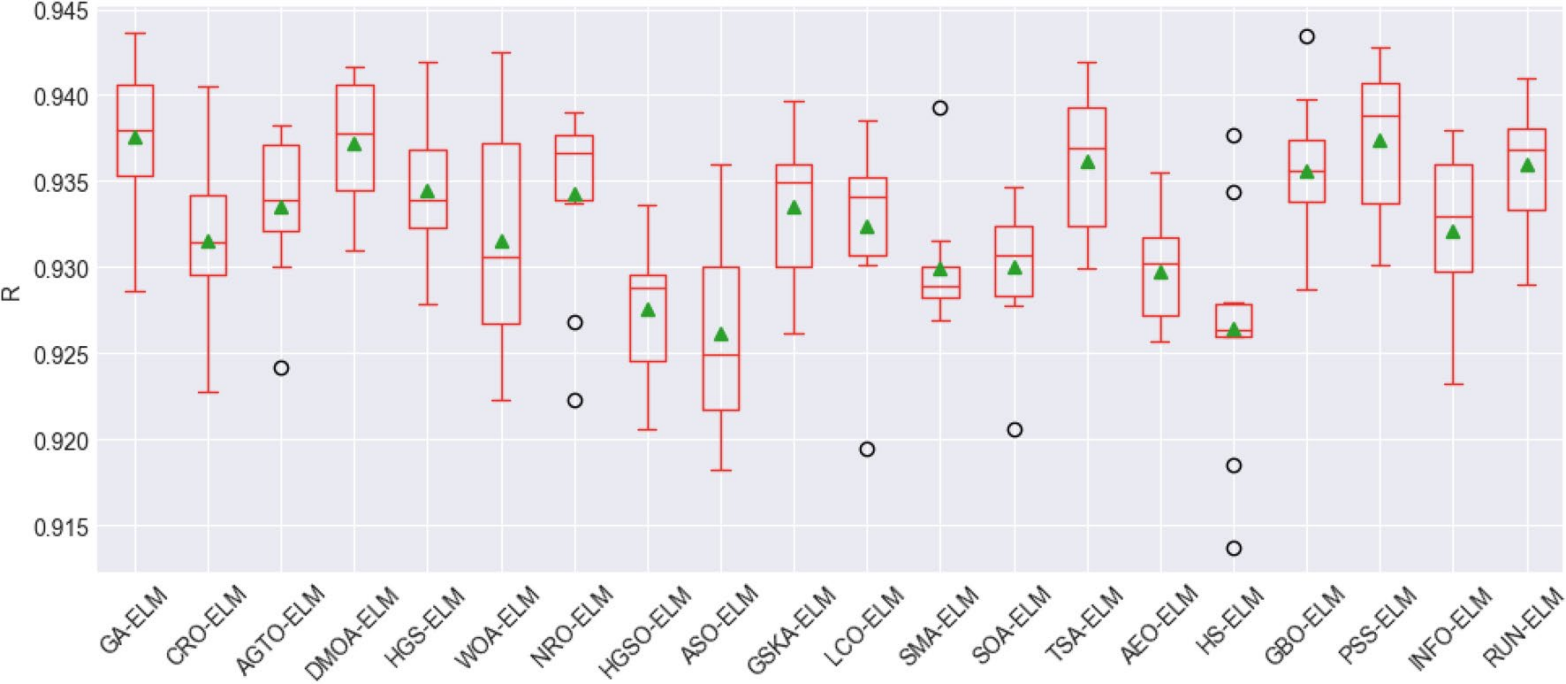
R results of all compared models after 10 trials (box plot).

**Figure 12:**
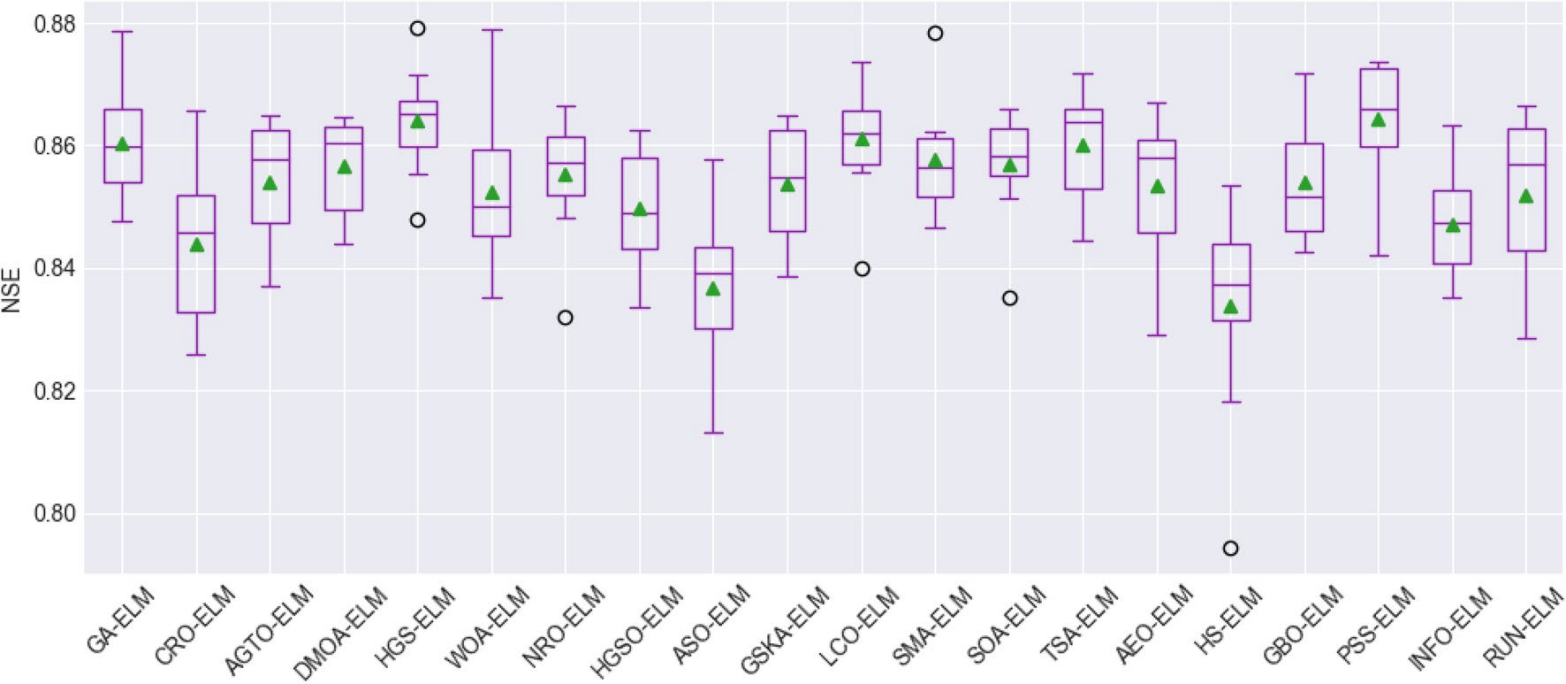
NSE results of all compared models after 10 trials (box plot).

**Figure 13:**
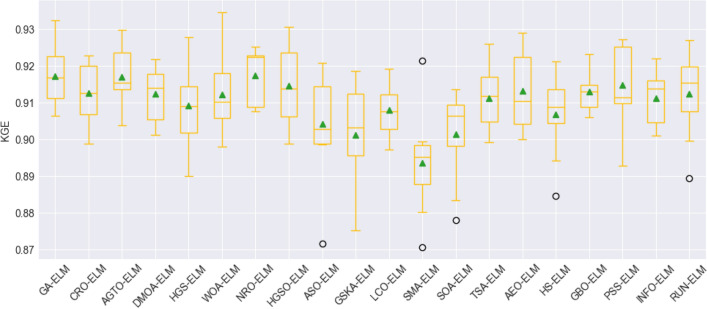
KGE results of all compared models after 10 trials (box plot).

To assess the stability and performance of the models, box plot visualization was used for the evaluation metrics, including the root mean square error (RMSE), mean absolute error (MAE), mean absolute percentage error (MAPE), Pearson's correlation coefficient (R), Nash–Sutcliffe efficiency (NSE), and Kling-Gupta efficiency (KGE). These figures, namely, Fig. 8, Fig. 9, Fig. 10, Fig. 11, Fig. 12, and Fig. 13, offer a visual representation of the results obtained from 10 independent trials of the model comparisons based on RMSE, MAE, MAPE, R, NSE, and KGE metric, corresponding. Box plots are a valuable tool in statistical analysis, providing a comprehensive view of the distribution and variability of the data. In each box plot, the box itself spans from the first quartile (Q1) to the third quartile (Q3) of the data, representing the middle 50% of the dataset. The line within the box denotes the median, which is the midpoint of the data series. Moreover, a green triangle symbol has been included within the box plots to indicate the mean value of each metric across the 10 trials. This offers a quick reference point for the central tendency of the results. It is important to note that the mean can be influenced by extreme values, so the box plot also highlights any outliers in the data.

Outliers, represented by hollow circles outside the main body of the box, are individual data points that fall significantly away from the majority of the dataset. These outliers, while not typical of the general trend, can provide valuable insights into unusual or extreme cases within the model performance. By presenting these box plots for each evaluation metric, a more comprehensive understanding of not only the central tendencies but also the variability and distribution of model performance across the trials has been introduced. This graphical representation allows for a nuanced comparison of the models, highlighting their strengths and weaknesses across a range of performance measures.

Overall, the use of box plots in this context provides a robust and visually intuitive method for assessing the stability, consistency, and overall performance of the compared models. These figures serve as a valuable resource for researchers and practitioners in making informed decisions regarding the selection and implementation of models for the studied application.

### Model convergence

**Figure 14:**
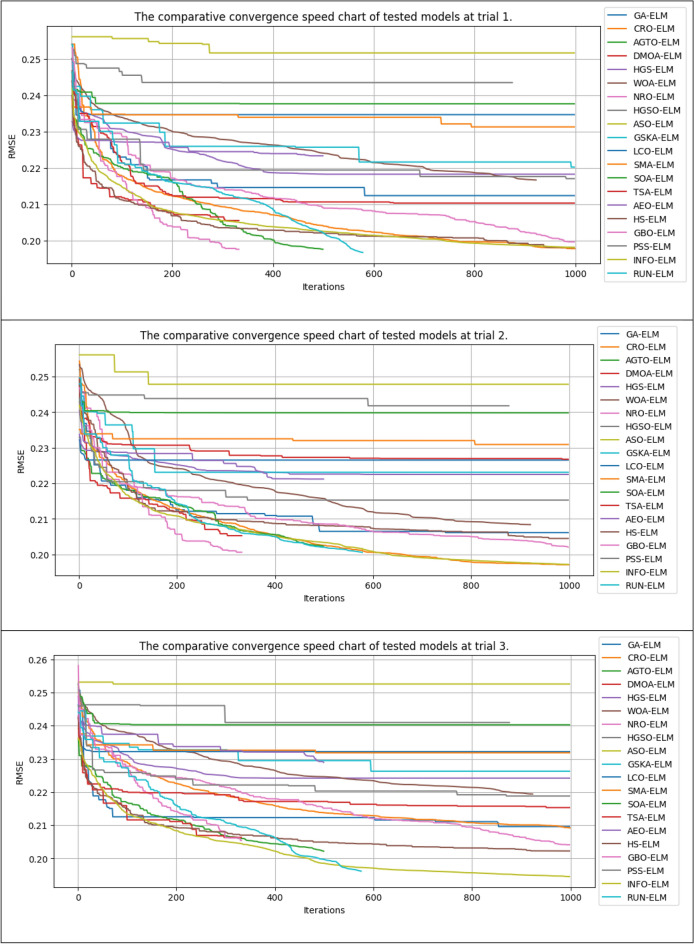

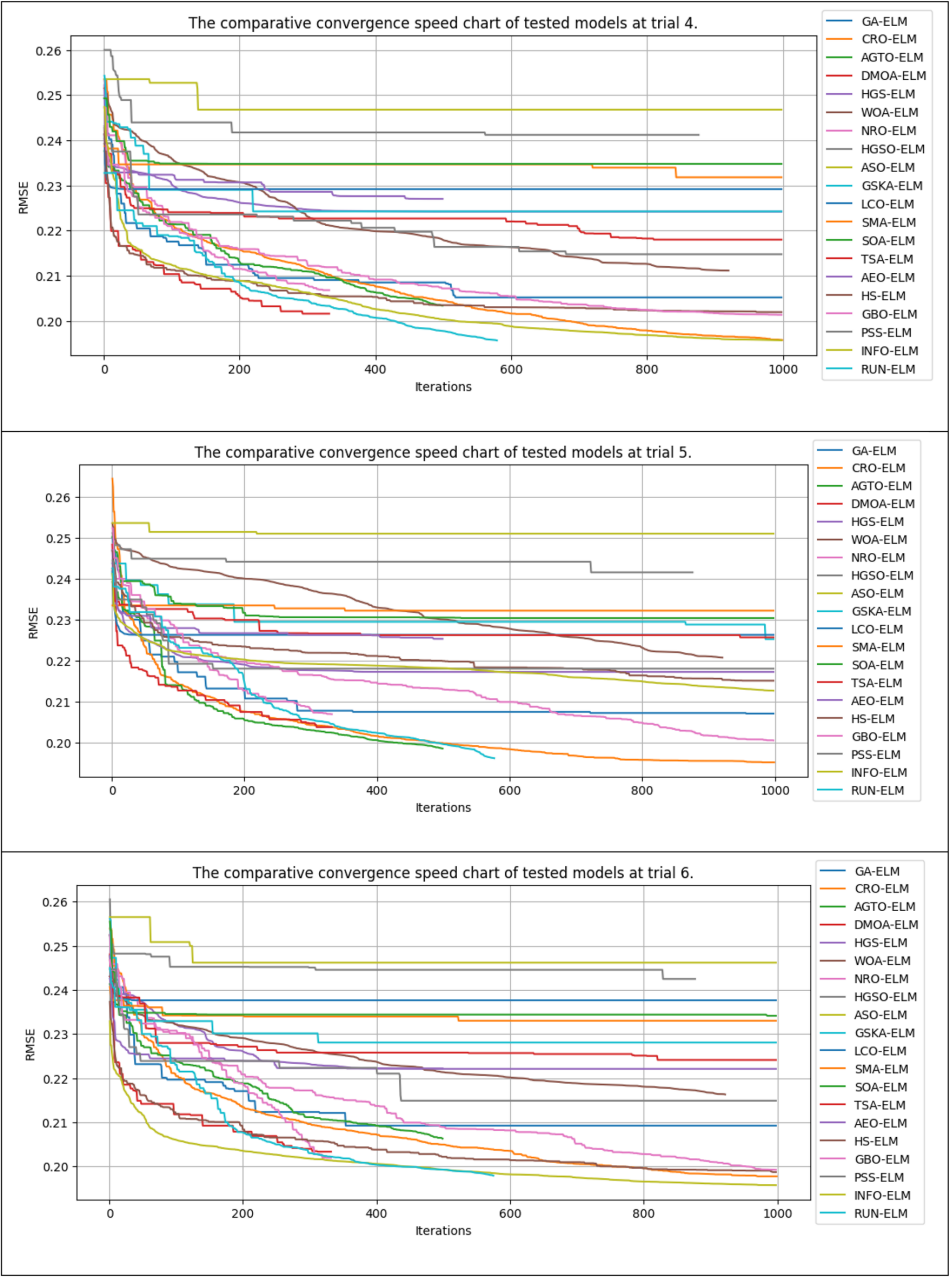

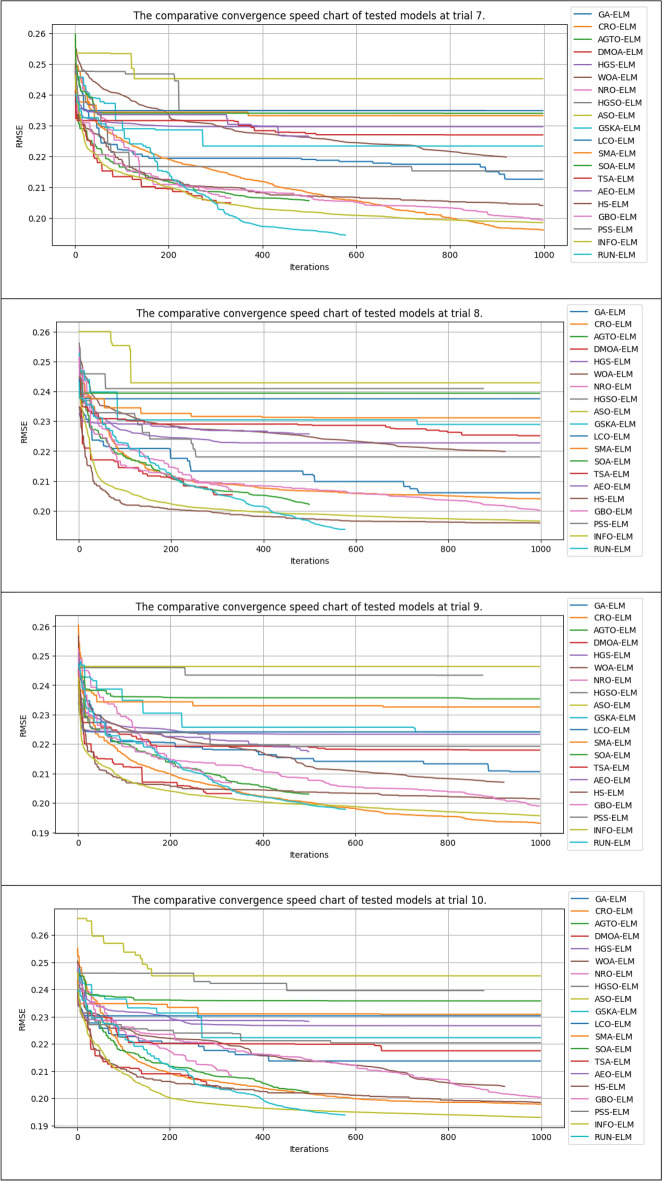
The convergence lines of the compared models in different trials.

This study also compared the model convergence during the training process. Figure 14 shows the error results after 50,000 NFE for the compared algorithms in several experimental runs. It should be noted that some algorithms in each iteration call the fitness function more than once for each search agent. Thus, some algorithms, such as the GSKA-ELM and NRO-ELM, execute fewer than 1000 iterations to reach the 50,000 NFE limit. In contrast, other algorithms that only call the fitness function once for each search agent in each iteration will perform 1000 iterations (50,000/50 = 10,000, population size = 50).

In general, our study revealed that the proposed INFO-ELM and RUN-ELM algorithms achieved more remarkable convergence than did the other algorithms. These algorithms achieved a very fast convergence speed after approximately 30 iterations. Although the convergence speed subsequently decreased, it still had the potential to continue decreasing rather than plateauing, similar to other algorithms such as ASO-ELM, HGSO-ELM, and CRO-ELM.

### Results comparison with previous study

Finally, we compare the performance of the proposed PSS-ELM model against established models, particularly the MLP model integrated with various physics-inspired metaheuristic algorithms as explored in^43^. Among these algorithms, Nuclear Reaction Optimization (NRO) exhibited promising results with an MLP model in forecasting streamflow, yielding RMSE = 2.35, MAE = 1.356, and MAPE = 16.747. However, our proposed PSS-ELM model surpasses the performance of NRO-MLP, achieving notably higher accuracy levels with RMSE = 2.0667, MAE = 1.2127, and MAPE = 0.1660. It can be seen that the improvement in MAPE from the NRO-MLP model to the proposed PSS-ELM model is substantial, reducing from 16.747 to 0.1660%.

In general, our proposed models (PSS-ELM, INFO-ELM, and RUN-ELM) offer valuable insights for decision-makers involved in water resource planning by the performance it demonstrates in terms of superior accuracy, stability, and convergence. The findings also highlight how the enhanced predictive capabilities of the proposed model can lead to more informed and effective management strategies, thereby mitigating the impacts of extreme events.

## Limitations and future directions

While our proposed PSS-ELM, INFO-ELM, and RUN-ELM models demonstrate promising performance in river streamflow prediction, it's essential to acknowledge potential limitations and biases that could impact their predictions and practical applications.

- Data limitations: One potential obstacle is the quality and quantity of input data. Despite efforts to use comprehensive streamflow data from the Aswan High Dam, historical data at monthly intervals spanning 130 years may miss short-term variations or localized phenomena. This constraint could affect the models' capacity to reliably capture quick changes or intense events, especially in places with limited data availability.
- Model assumptions: Our model, like other modeling methods, uses metaheuristic algorithms, thus some parameters must be calibrated. Assumptions about these features may be good for this case study but not for others, especially for data types with complex relationships and nonlinear dynamics found in hydrological systems. Biases towards parameters that are beneficial to the model might cause disparities between model predictions and observed results. Furthermore, ELM-based models are shallow neural networks with a simplistic structure that may fail to capture complex correlations in hydrological data with many constraints.
- Generalization to different conditions: Another potential limitation is the generalizability of our models to other meteorological, geographical, and hydrological situations. While we validated our models using data from the Aswan High Dam, their performance in other areas with different hydrological regimes and environmental variables is unknown. Differences in climate, terrain, land use, and hydrological processes may add biases into model projections in practical applications outside of the research area, affecting their dependability and accuracy.

These limitations and biases may have significant implications for model predictions and their practical applications in water resource management and flood risk mitigation, such as:

- Prediction uncertainty: Biases caused by data restrictions and model assumptions may inject uncertainty into model predictions, especially during extreme events or in non-stationary environments. Decision-makers who rely on these estimations may need to assess the related uncertainty and develop risk-mitigation techniq
- Misinterpretation risk: Biases caused by model constraints may result in misinterpretation of outcomes or overreliance on predictive models during decision-making processes. To enable informed decision-making, stakeholders must be made aware of the models' inherent uncertainties and limits.
- Transferability problems: Generalizing model predictions to different scenarios or places may be difficult due to biases caused by changes in environmental variables. Careful validation and adaption of the models to unique circumstances are required to assure their dependability and usability in practical applications beyond the study domain.

Considering the limitations mentioned above, future directions could focus on various areas to increase the robustness and practical use of the models:

- Parameter Optimization: Develop methods for optimizing model parameters so that they are more robust and adaptive to changing conditions, taking into account the complex interactions and nonlinear dynamics found in hydrological systems.
- Model Generalisation: Research methods aimed at improving the models' generalization capability across a wide range of climatic, geographic, and hydrological scenarios. This could require incorporating more diverse datasets and accounting for fluctuations in environmental variables.
- Regional Validation: Thoroughly validate the models in locations with different hydrological regimes and environmental factors to assess their performance and reliability beyond the original study area.
- Enhanced Model Complexity: Investigate the possible advantages of increasing model complexity, such as utilizing more advanced neural network designs or incorporating extra environmental variables, in order to better depict the complexity of hydrological systems.

## Conclusion

In this study, novel models combining three advanced math-inspired metaheuristics: Pareto-like sequential sampling (PSS), weighted mean of vectors (INFO), and Runge–Kutta optimization (RUN) with an extreme learning machine (ELM) for streamflow forecasting tasks are introduced as effective approaches. These approaches address the limitations of random weights in the matrix H of the ELM network, which are set once during training and remain unchanged. Consequently, relying solely on the Moore–Penrose inverse operation may lead to suboptimal solutions if the initial weights do not represent the global optimum. To mitigate this challenge, new algorithms such as PSS, INFO, and RUN, known for their gradient-free nature, capacity to escape local optima, and global optimization capabilities, have recently been developed.

To assess the performance of these hybrid methods, 20 models have been evaluated across eight primary metaheuristic categories: evolutionary-inspired, swarm-inspired, human-inspired, physics-inspired, music-inspired, biology-inspired, system-inspired, and math-inspired. Utilizing a dataset of streamflow data from the Aswan High Dam (AHD) spanning 130 years at monthly intervals—a crucial resource for water management. Our findings indicate that the prediction accuracy, model convergence, and stability of the ELM combined with math-inspired methods surpassed those of other methods. Specifically, our proposed PSS-ELM model demonstrated superior accuracy in river streamflow prediction with a Pearson’s correlation index (R) of 0.9374, root mean square error (RMSE) of 2.0667, and a Nash–Sutcliffe efficiency (NSE) of 0.8642. Furthermore, the INFO-ELM and RUN-ELM models exhibited enhanced stability across ten runs, showing lower mean absolute percentage errors (MAPE) of 15.21% and 15.28%, respectively, as well as better values of mean absolute errors (MAE) with 1.2145 and 1.2105, respectively, and higher values of Kling-Gupta efficiencies (KGE) at 0.9113 and 0.9124, respectively. These results underscore the potential effectiveness of our proposed PSS-ELM, INFO-ELM, and RUN-ELM models in natural streamflow forecasting systems.

While our models demonstrated robust performance in river streamflow forecasting, it is crucial to acknowledge that this study represents a single case under specific climatic conditions. Further investigations across diverse climatic scenarios and regions are essential to comprehensively evaluate the models' effectiveness. Thus, our future research will aim to apply these models to various case studies characterized by distinct climatic conditions, broadening the scope of our analysis in water resource-related applications. Besides, future work can be carried out by the deployment of the developed model in real-time forecasting scenarios. This will further demonstrate the practical utility and effectiveness of our approach in addressing real-world challenges in water resource management.

## Data Availability

In this study, all of the performance metrics are taken from https://github.com/thieu1995/permetrics. All of the metaheuristic algorithms are taken from https://github.com/thieu1995/mealpy. The materials and models are available at https://github.com/aiir-team/math-MHA-ELM-streamflow-code. The dataset for this study is available from the fourth author (Ahmed El-Shafie, elshafie@um.edu.my) by reasonable request.
